# Uncovering the Relationship between Tissue-Specific TF-DNA Binding and Chromatin Features through a Transformer-Based Model

**DOI:** 10.3390/genes13111952

**Published:** 2022-10-26

**Authors:** Yongqing Zhang, Yuhang Liu, Zixuan Wang, Maocheng Wang, Shuwen Xiong, Guo Huang, Meiqin Gong

**Affiliations:** 1 School of Computer Science, Chengdu University of Information Technology, Chengdu 610225, China; 2 School of Electronic Information and Artificial Intelligence, Leshan Normal University, Leshan 614000, China; 3 West China Second University Hospital, Sichuan University, Chengdu 610041, China

**Keywords:** TF-DNA binding, chromatin features, tissue specific, deep learning

## Abstract

Chromatin features can reveal tissue-specific TF-DNA binding, which leads to a better understanding of many critical physiological processes. Accurately identifying TF-DNA bindings and constructing their relationships with chromatin features is a long-standing goal in the bioinformatic field. However, this has remained elusive due to the complex binding mechanisms and heterogeneity among inputs. Here, we have developed the GHTNet (General Hybrid Transformer Network), a transformer-based model to predict TF-DNA binding specificity. The GHTNet decodes the relationship between tissue-specific TF-DNA binding and chromatin features via a specific input scheme of alternative inputs and reveals important gene regions and tissue-specific motifs. Our experiments show that the GHTNet has excellent performance, achieving about a 5% absolute improvement over existing methods. The TF-DNA binding mechanism analysis shows that the importance of TF-DNA binding features varies across tissues. The best predictor is based on the DNA sequence, followed by epigenomics and shape. In addition, cross-species studies address the limited data, thus providing new ideas in this case. Moreover, the GHTNet is applied to interpret the relationship among TFs, chromatin features, and diseases associated with AD46 tissue. This paper demonstrates that the GHTNet is an accurate and robust framework for deciphering tissue-specific TF-DNA binding and interpreting non-coding regions.

## 1. Introduction

Transcription factors (TFs) are proteins with special functions that can control transcription by binding to DNA sequences, thereby regulating gene expression [1,2]. Their binding sites are known as TFBSs. However, TF-DNA binding relates not only to the conserved DNA sequence preferences but also to DNA shape and chromatin state [3,4,5]. These TF-DNA binding features are essential for characterizing where TFs bind to the genome and for further understanding how TFs control gene expression. Numerous studies have confirmed that TFs play a crucial role in human physiological processes due to their extensive tissue-specific binding [6,7]. Therefore, uncovering the mechanism of tissue-specific TF-DNA binding is essential for studying how TFs are involved in transcriptional regulation, exploring gene functions, and understanding cellular activities in different tissues [8,9].

Many TF-DNA binding studies have previously been conducted; they have shifted from traditional biological experiments to machine learning (ML) and then to deep learning (DL) [10]. DeepBind [11] and DeepSEA [12] pioneered the use of the convolutional neural network (CNN) to predict TF-DNA binding specificity. After that, many improved methods have been proposed, mainly divided into two categories: CNN-based and CNN+ recurrent neural network (RNN)-based [13]. (i) CNN-based methods mainly include dilated CNN [14], fully CNN [15], and residual CNN [16]. (ii) CNN+RNN-based methods mainly include hybrid CNN with long short term memory (LSTM) [17] and gated recurrent units (GRU) [18]. CNN-based methods can learn the spatial dependence of TF-DNA binding motifs, and CNN+RNN-based methods can capture the long-range dependence. Although many computational methods have been proposed, the complex mechanisms of TF-DNA binding do not have clear genetic codes, leading to incomplete elucidation when using DNA sequence alone.

A more comprehensive view of biological processes can be obtained through an integrated analysis of different chromatin features [19]. Previous studies have shown that TF-DNA binding is not only related to a DNA sequence, but also to various other factors, such as DNA shape [20], histone modification (HM) patterns surrounding TFBSs [21], and chromatin accessibility [14]. Studies on DNA shape have shown that combining DNA shape with the sequence can effectively improve the predictive power of TFBSs [22,23]. Furthermore, the motif identification methods based on DNA shape have also achieved excellent success [24]. Studies on HM patterns have shown that the level of HMs varies in different tissues. Using HM patterns may also improve the predictive power of the model [25]. Simultaneously, numerous studies of TFs binding using chromatin accessibility have been conducted [26]. In addition, the conservation score reflects the conserved nature of each position on the human genome, and highly conserved DNA sequences may have a functional value. However, due to the high-dimensional heterogeneity of these features, how to represent and fuse them is still a problem. Moreover, most current studies have used these features to enhance model performance. Still, they have not systematically analyzed the relationship between tissue-specific TF-DNA binding.

The research on tissue-specific TF-DNA binding still has the following problems: (i) Constructing a TF binding prediction model based on different DNA binding features is challenging. Most current methods are based on DNA sequence features because if the high-dimensional heterogeneity of their various features. (ii) The relationship between tissue-specific TF-DNA binding and epigenomics is still unclear. The research on TF-DNA binding at the tissue level is minimal. Most previous research has focused on a specific chromatin feature. They did not systematically analyze the impact of different factors on TF-DNA binding. (iii) The application scenarios of current models are still insufficient. Most existing models only predict the TF-DNA binding sequence and do not perform any further analysis.

Since 2017, transformer [27] has gradually become the primary trend in natural language processing (NLP) due to its excellent performance when using the self-attention mechanism, which outperforms both CNN- and RNN-based models. In addition, the self-attention mechanism is inherently interpretable and allows for parallel computation. Importantly, transformer is ideally suited for multimodal applications because its self-attention structure can adapt to various data types [28]. Therefore, it has received extensive attention in various fields. In bioinformatics, researchers usually divide the genome into *k*-mer segments to obtain higher-order dependencies, which are an analogy to words in a text [29]. This genome characteristic makes it possible to extract information from biological sequences similar to NLP. To transform the high-dimensional one-hot encoding into a dense vector, the word2vec technique was first proposed to learn distributional vector embeddings. Recently, transformer-based models have been used to predict protein structures by researchers at Google [30], and other researchers have used transformer-based models to interpret gene regulation [31], which have both led to new advancements in the bioinformatics field.

**Figure 1:**
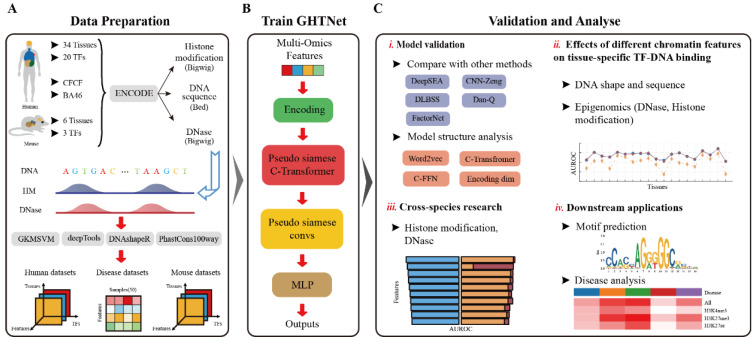
Overview of the GHTNet framework and validation. (**A**) Multiple raw data in different tissues of humans and mice are collected from ENCODE. These raw data are processed using GKMSVM [32], deepTools [33], DNAshapeR [20], and phastCons100way [34]. Three datasets are generated for training GHTNet, including human, disease, and mouse datasets. (**B**) The Overview architecture of our model, predicting gene transcription binding using multiple TF-DNA features. (**C**) The four main analyses of this study.

Motivated by these previous studies and observations, we thus propose a transformer-based model to solve these existing problems. Our model utilizes self-attention and CNN to predict TF-DNA binding specificity and is named the GHTNet. A word embeddings (word2vec) strategy based on *k*-mer is employed to encode DNA sequences. Additionally, the multi-head self-attention mechanism and convolution-FFN (C-FFN) are used to capture the global dependencies. Finally, CNN is used to extract the low-level features of TFBSs, and multilayer perceptron (MLP) is used to identify high-level features after CNN is completed. Using this model, we investigated the TFs tissue specificity, focusing on the importance of different TF-DNA binding features in various tissues. Furthermore, taking advantage of the interpretability of the GHTNet, important gene regions and tissue-specific motifs were identified. Cross-species experiments were conducted to verify the high degree of conservation between humans and mice, which provides new ideas about the limited available data. Finally, the relationships among TFs, chromatin features, and diseases associated with middle frontal area 46 (AD46) tissue were analyzed. See Figure 1 for an overview.

In summary, the main contributions of this article are three-fold: (i) This paper proposed a transformer-based TF-DNA binding prediction model, which can integrate multiple chromatin features, has good interpretability, and can identify motifs. (ii) This paper studied the effect of different chromatin features on tissue-specific TF-DNA binding, elucidating the relationship between tissue-specific TF-DNA binding and chromatin features. Additionally, we further conducted transfer learning to study the feasibility of cross-species experiments. (iii) This paper conducted downstream analysis by extending GHTNet. We predicted the TF-DNA binding motifs, inferred the important gene regions, and further analyzed the relationship between TFs, HM patterns, and diseases.

## 2. Materials and Methods

### 2.1. Data Preparation

Five TF-DNA binding features in genomics and epigenomics were used in this study, including DNA sequence, DNA shape, HM pattern, chromatin accessibility, and conservation score. In the following, we briefly describe the process of data preparation. 

DNA sequence: Chromatin immunoprecipitation followed by sequencing (ChIP-seq) data from ENCODE [35] was collected for all human and mouse tissues. In addition, 50 datasets of CTCF in human AD46 tissue were collected for disease analysis. GKM-SVM [32] was used to generate DNA sequences for processing these data. The gene coordinates centered on the ChIP-seq peaks for each dataset were expanded to 101 bp as the positive sequence. Simultaneously, the regions with similar GC content to positive sequences were selected as negative sequences from the whole genome. Finally, we obtained a dataset with an equal number of positive and negative samples.

DNA shape: DNAshapeR [20] was used to generate 13 DNA shapes and electrostatic potential (EP) of each positive and negative sequence. DNA shapes were obtained by Monte-Carlo simulation, which was calculated by a slide window method. According to its structure, it can be divided into three categories, (i) inter-bp: HelT, Rise, Roll, Shift, Slide, and Tilt; (ii) intra-bp: Buckle, Opening, ProT, Shear, Stagger, and Stretch; and (iii) MGW and EP. Since the values of different shapes vary greatly, all the results were normalized to [1].

Epigenomics: HM patterns and chromatin accessibility were used in our experiment, including two HM patterns, H3K27me3 and H3K9me3, and DNase I hypersensitive site (DNase-seq). We collected all human tissue data from ENCODE. Moreover, HM patterns from 50 human BA46 tissues were collected, including H3K27ac, H3K27me3, and H3K4me3. In addition, all HM patterns common to humans and mice were collected for cross-species studies. Then, deepTools [33] was used to process raw data, constructing base-resolution feature vectors. 

Conservation score: This score was calculated based on comparing the human genome with the genomes of 99 other vertebrates. It reflects the conserved regions in biological evolution, which may be potential gene functional regions. PhastCons100way [34] generated conservation scores for each sequence, which is an additional model feature.

Due to the absence of some features, after removing the datasets with missing features, we divided these datasets into three categories according to the research content. It consisted of three datasets, 86 human datasets with 20 TFs in 34 tissues, 9 human and mouse datasets with 3 TFs in 6 tissues, and 50 disease datasets with CTCF in human AD46 tissue.

### 2.2. Overview of the GHTNet Architecture

**Figure 2:**
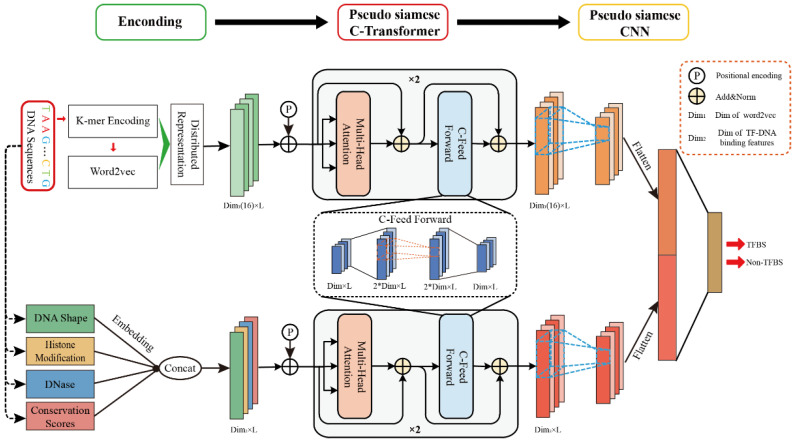
The architecture of the proposed model the GHTNet. It consists of three modules, including encoding, pseudo Siamese C-transformer, and pseudo Siamese CNN module. Different encoding methods are used to encode the data in the encoding module. Pseudo Siamese C-transformer module is used to establish the dependencies between the input features. Pseudo Siamese CNN module is used to make classification.

The architecture of our proposed model the GHTNet is shown in Figure 2, which consists of three modules: encoding module, pseudo Siamese C-transformer module, and pseudo Siamese CNN module. The pseudo Siamese C-transformer module is our model’s core component, consisting of a multi-head self-attention layer and a C-FFN. The convolutional position-wise feed-forward network is constructed by adding a CNN in the position-wise feed-forward network (FFN). With these modules, we can realize TF-DNA binding specificity prediction based on multi-data. In the following, we briefly introduce these three modules, and the detail can be found in Supplemental Materials.

#### 2.2.1. Encoding Module

This study used five kinds of data: DNA sequence, shape, histone modification, DNase, and conservation score. Due to the heterogeneity between different features, encoding them is challenging. For example, DNA sequences are discrete characters (i.e., A, G, C, T, and Unknown), and DNA shapes are discrete vectors that can be calculated by the slide window method. In contrast, HMs and DNase are formatted as a ‘bigwig’ compressed file, which is helpful for dense and continuous data. To address these issues, different encoding strategies were applied to DNA sequence and TF-DNA binding features. For DNA sequence, it was divided into *k*-mer segments to fully consider the high-order dependency. To ensure the sequence length was consistent, character ‘N’ is padded before and after the sequence. For example, a DNA sequence “ATCGA” can be tokenized to five segments with 3-mers (NAT, ATC, TCG, CGA, GAN). Due to the GHTNet requiring binary vectors as input, a one-hot encoding strategy was used to represent each segment. Since five characters may occur at each position, the encoding dimension was 5*^k^*, causing a high-dimensional sparsity problem. Therefore, the word2vec strategy was used to learn low-dimensional representations of segments. Specifically, 3-mer with 1-stride settings were generated to tokenize the sequences. Finally, the high-dimensional one-hot encoding segments were converted to *n* × *d* low-dimensional distributed representation matrix, where *n* = 101, *d* represents the dimension of the distributed representation (Supplementary Figure S12). For other TF-DNA binding features, it was directly generated from preprocessed raw data.

#### 2.2.2. Pseudo Siamese C-Transformer Module

Then, we leveraged the encoder part of the transformer architecture, which consisted of a multi-head self-attention layer and an FFN. Meanwhile, the residual module was added to them, followed by layer normalization. The multi-head self-attention layer can adaptively identify important gene regions, and FFN realizes the purpose of space transformation. To better focus on the TFBSs, a convolution operation was added to the FFN, which is called C-FFN. Previous studies have shown that DNA sequences are an essential feature. Thus, a pseudo Siamese network was constructed to deal separately with DNA sequence and other TF-DNA binding features.

#### 2.2.3. Pseudo Siamese CNN Module

CNN was used to extract TF-DNA binding features (motifs), followed by a max pooling layer to reduce computational consumption. We constructed a pseudo Siamese network for DNA sequence and TF-DNA binding features to handle the different data types separately. The concatenation of the output from the max pooling layer is fed into a fully connected layer to realize TF-DNA binding specificity. 

### 2.3. Model Training

Two models needed to be trained in this study. The word2vec model captures high-order dependency and the GHTNet is used to predict the TF-DNA binding specificity.

#### 2.3.1. Word2vec Model Training

Word2vec is one of the commonly used models in NLP, which can learn semantic knowledge in an unsupervised manner from a large amount of text. There are two strategies to train a word2vec model, predicting neighboring words using a given center word (CBOW model) and predicting a center word using given neighboring words (Skip-gram model) [36]. Because Skip-gram contains more knowledge about the context, we employed the Skip-gram strategy to obtain high-order dependency (Supplementary Figure S1). To tradeoff performance and computational cost, we randomly sampled 5000 sequences from each positive dataset, constituting a training set of about 430,000 sequences to train the word2vec model. In particular, all sequences were used for datasets with less than 5000 samples. The objective function *L_w_* can be expressed as:

$$L_{w}=\sum logp(context\left(w\right)|w)$$

where multilayer perceptron (MLP) is used to predict the *context*(*w*) of a given center word *w*, where *w* represents the input word, and *context*(*w*) represents words around *w*. The loss function was optimized by mini-batch gradient descent with batch size = 256. Adam was used as an optimizer with a learning rate = 0.001. Due to the computational volume and representation utility, three types of distributed representation dimension (dimension = 8, 16, 32) and five type of *k*-mer (*k* = 2, 3, 4, 5, 6) were generated. After the experiments, we chose distributed representation dimension = 16 and *k* = 3 as the final settings. The training was stopped when the loss no longer decreases. Finally, the hidden layer’s weight matrix can be defined as the distributed representation of the segments.

#### 2.3.2. GHTNet Training

Each dataset was divided into a training set, validation set, and test set, accounting for 80%, 10%, and 10%, respectively. Batch optimization and cross-entropy are utilized:

$$L=\frac{1}{N}\overset{N}{\underset{i=1}{\sum }}\left(y_{i}\log{\hat{y}}_{i}+\left(1-y_{i}\right)\log\left(1-{\hat{y}}_{i}\right)\right)$$

where *L* represents the average loss of a batch, *N* represents the batch size, and *y_i_* and ${\hat{y}}_{i}$ represent the ground-true and predicted probability for each of *N* classes. AdamW optimizer was used to optimize the model based on Adam+L2 regularization:

AdamW(Θ,grad(L))

where *grad*(*·*) represents gradient descent, and Θ refers to the model parameters. For hyperparameter selection, the mini-batch size was 64; the dropout rate was 0.2. The learning rate adopted the warm-up strategy, where the starting value is 1 × 10^−4^ and increases linearly to 5 × 10^−4^ after ten epochs. For the model structure, the stacking number of self-attention modules *L* = 2. For model training, the weight decay was 0.01, and beta1 and beta2 were 0.9 and 0.99, respectively. An early stopping strategy was used to prevent overfitting, where the training is stopped after the loss of the validation set does not drop within five consecutive epochs. Five-fold cross-validation was used, and the average is taken as the final result. This experiment implemented the model in the Pytorch environment, and Nvidia 3080 was used to train the model. The algorithm flow can be found in Supplementary Text S2.

### 2.4. Comparison with Five TF-DNA Binding Prediction Methods

We compared our model with three classes state-of-the-art TF-DNA binding prediction methods: (i) DNA sequence-based methods, such as DeepSEA [12], DanQ [17], and CNN_Zeng [37]; (ii) a DNA sequence+shape-based method, such as DLBSS [38]; and (iii) a DNA sequence+epigenomics-based method, such as FactorNet [26]. For a fair comparison, all methods had the same optimizer and hyperparameter settings. Five-fold cross-validation was used to reduce bias. A brief introduction to the comparison method is as follows.

DeepSEA is one of the earliest models to predict TFBSs using DL, which mainly includes convolutional layers, max-pooling layers, and fully connected layers. CNN_Zeng is similar to DeepSEA, with improvements in model parameters, including convolutional layers, max-pooling layers, and fully connected layers. DanQ is an improvement on DeepSEA. It includes a convolutional layer, max-pooling layer, bidirectional LSTM layer, and fully connected layer. DLBSS uses shared convolution to capture shared features in DNA sequences and shapes and then uses fully connected layers to obtain final results based on the output from the CNN. FactorNet combines DNase feature with DNA sequence to construct a pair of Siamese networks finalized based on positive and negative strand information. It includes convolutional layers, max-pooling layers, bidirectional LSTM, and fully connected layers.

Three metrics were used to evaluate the model performance: accuracy, AUROC, and AUPRC. The *t*-test was used to find the corresponding *p*-value if the variance between samples was the same; otherwise, the Wilcoxon test was used. A detailed description of these metrics can be found in Supplementary Text S1.

## 3. Results

### 3.1. Model Validation

#### 3.1.1. GHTNet Achieves Superior Performance by Integrating Different Chromatin Features

TF and their corresponding DNA shapes, HM patterns, DNase, and conservation scores were collected from ChIP-seq and DNase-seq data. We obtained 86 datasets with 20 TFs in 34 tissues. Then, we compared GHTNet with five state-of-the-art methods: DeepSEA, CNN_Zeng, DanQ, DLBSS, and FactorNet. The results show that GHTNet achieves Acc of 92.79%, yielding extra 8.29%, 8.02%, 7.92%, 8.49%, and 6.38% improvements over CNN_Zeng, DanQ, DeepSEA, DLBSS, and FactorNet, respectively (Table 1). Likewise, our model has the best average performance according to AUROC and AUPRC compared to the other methods (Supplementary Figure S3).

We also observed that the CNN+RNN-based model performed better than the CNN-based model, which is consistent with the results of previous studies. However, the existing CNN+RNN-based model just slightly improved the performance. For example, DanQ achieves AUROC 0.9121, yielding extra 0.0041 (*p* = 2.30 × 10^−5^) and 0.0035 (*p* = 3.10 × 10^−4^) improvements over DeepSEA and CNN_Zeng, respectively. To make a fair comparison with other DNA sequence-based methods, we constructed GHTNet-DNA (Supplementary Figure S2), which only relies on the DNA sequence. The results show that GHTNet-DNA outperformed the other DNA sequence-based methods (AUROC, *p* < 0.05). We also compared it with transformer, and the results show that the GHTNet outperforms transformer by a large margin (Supplementary Figure S13). This demonstrates the excellent performance and robustness of the GHTNet, not only due to data fusion but also because of its model structure.

We investigated the relationship between TF-DNA binding and its binding features in different tissues. The results suggest that the importance of these TF-DNA binding features varies across tissues (Supplementary Table S2). For example, the prediction results of GHTNet-DNA for CTCF were significantly higher than PLOR2A (AUROC, *p* = 9.70 × 10^−3^). However, when multiple TF-DNA binding features were fused, these two TFs performed similarly (AUROC, *p* = 0.86). This finding implies that other conserved patterns, such as DNA shape and HM patterns, may also involve TF-DNA binding recognition. In addition, we found that DNA sequence has different importance for different tissue. Overall, the experiments demonstrate that the GHTNet has excellent performance and can be further used to explain the significance of TF-DNA binding features.

#### 3.1.2. GHTNet Has the Optimal Structure

Model ablation and parameter tuning were conducted to understand the effects of model structure and hyperparameters on TF-DNA binding specificity. In the following experiments, one variable was changed simultaneously with all other parameters set to default values to control computational cost. All experiments were repeated with five-fold cross-validation to reduce the bias caused by dividing the dataset.

**Figure 3:**
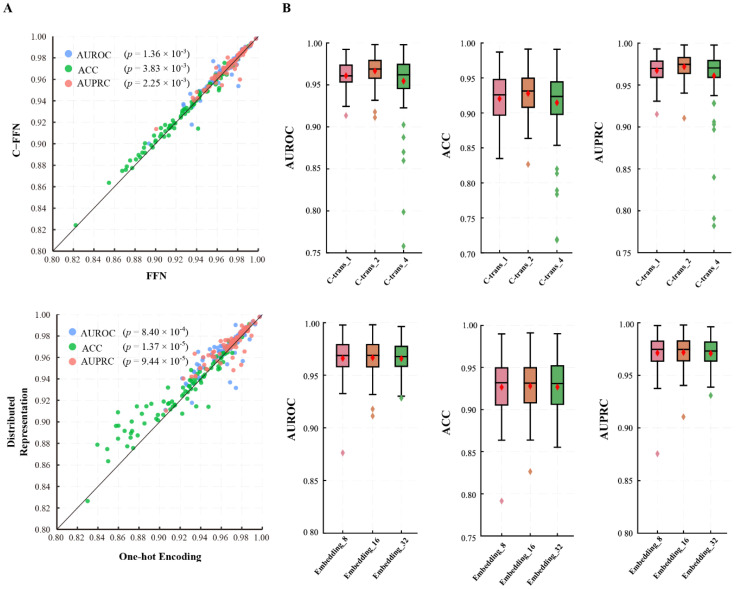
Performance comparison of different parameters setting. (**A**) The upper and lower scatter plots represent the results of the ablation of the C-FFN and word2vec modules, respectively. (**B**) The upper and lower boxplots represent the AUROC, ACC, and AUPRC of the GHTNet by varying the number of C-transformer layers and the embedding dimension, respectively.

Firstly, ablation experiments were performed on the first dataset to check the effectiveness of different configurations of the GHTNet (Figure 3A). We mainly tested two model configurations, without word2vec strategy and CNN in C-FFN. The first model replaced the word2vec module with a one-hot encoding model. When the word2vec module was removed, the performance decreased slightly, with the difference of only 0.0036 (*p* = 8.40 × 10^−4^), 0.0066 (*p* = 1.37 × 10^−5^), 0.0033 (*p* = 9.44 × 10^−5^) in AUROC, ACC, and AUPRC, respectively. For the second model, FFN was deployed in the encoder instead of C-FFN. Experiments show that after we replaced the module, the AUROC, ACC, and AUPRC decreased by 0.0019 (*p* = 1.81 × 10^−3^), 0.0015 (*p* = 7.36 × 10^−3^), and 0.0013 (*p* = 4.27 × 10^−5^), respectively. In conclusion, although the performance gains from these modules are modest, these modules play an important role in TF-DNA binding specificity prediction.

Next, hyperparameter selection experiments based on the optimal model structure were performed to investigate the robustness of the model (Figure 3B). Firstly, 8, 16, and 32 for the encoding dimensions of the word2vec distributed representation were chosen for the experiments. The results show that the encoding dimension had less influence on the model. Specifically, the ACC improved by 0.13% (*p* = 8.66 × 10^−2^) when the coding dimension was increased from 8 to 16, but also had a similar performance when it was further increased to 32. Since the model parameters increased significantly when the encoding dimension was 16 to 32, we chose the encoding dimension to be 16. Then, we experimented with the number of encoder layers, testing one, two, and four layers to find the optimal setting. The results show that the model was optimal when the number of layers equaled two. The AUROC is improved 0.0057 (*p* = 2.00 × 10^−3^) and 0.012 (*p* = 9.26 × 10^−8^) compared with the one-layer and four-layer models. Moreover, five types of *k*-mer (*k* = 2, 3, 4, 5, 6) strategies were evaluated. We hypothesize that increasing the information content can better represent regulatory elements such as motifs. For all *k*-mer settings, the GHTNet achieved excellent performance, outperforming the one-hot encoding strategy, and thus proving the robustness of the model (Supplementary Table S3). However, the model performance is optimal at *k* = 3, which is inconsistent with our hypothesis. This may be caused by challenging high-dimensional features, insufficient sample size, and increased model parameters. Through the above experiments, we demonstrated the robustness of the GHTNet.

### 3.2. Effects on Different Chromatin Features on Tissue-Specific TF-DNA Binding

#### 3.2.1. DNA Sequence and Shape Are Crucial Factors Affecting Tissue-Specific TF-DNA Binding

Previous studies have shown that DNA 3D structures are essential for predicting TF-DNA binding. However, there are few studies on the importance of DNA shape in different tissues. To investigate this, we extended the GHTNet to interpret the contribution of DNA shape and elucidate TF-DNA binding specificity.

**Figure 4:**
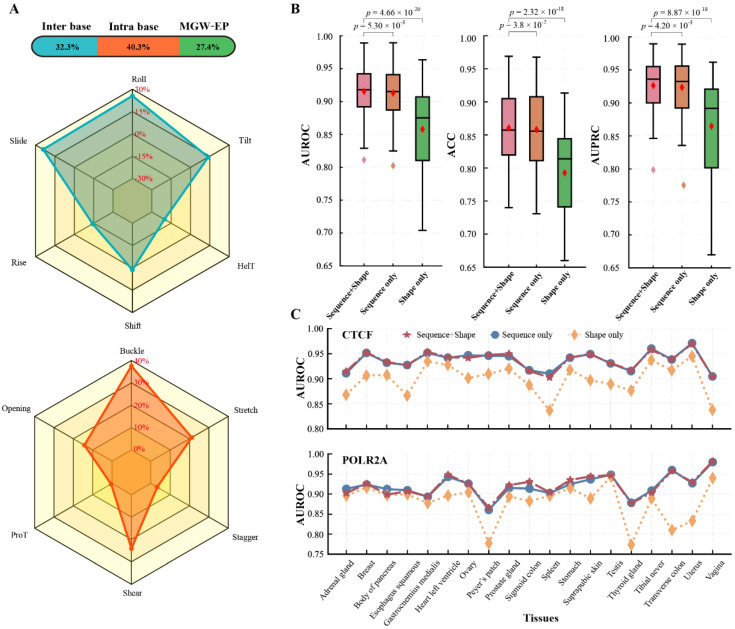
Importance analysis of DNA sequence and shape. (**A**) The contribution of DNA shape in predicting TF-DNA binding specificity. The upper figure shows the contribution of three types of DNA shapes. The middle and lower figures show the contribution of each DNA shape in the inter-base and intra-base categories, respectively. (**B**) The AUROC, ACC, and AUPRC of different inputs based on the 86 human ChIP-seq datasets and *p*-value between different models. (**C**) The AUROC of CTCF and POLR2A in different tissues.

Firstly, we analyzed the effect of DNA shape on gene transcription binding prediction by using 13 DNA shapes and EP. The results demonstrated that these 14 features could predict TF-DNA binding. Still, the performance was lower than models using DNA sequences (Figure 4B). On average, AUROC decreased 0.055 (*p* = 3.06 × 10^−19^). Notably, for POLR2A in testis tissue, DNA shape is more important than the sequence. To avoid the impact of chance factors, we repeated the experiments on another dataset and obtained the same results. Although the cause is unknown, TFs can depend on DNA shape for tissue-specific binding.

In addition, we observed that the AUROC performance between sequence and shape has a weak positive correlation (Pearson *r* = 0.40). These results suggest that they do not match one-to-one. DNA shape mainly depends on DNA sequence, which means that the exact shape can be encoded by different DNA sequences [20]. To investigate the relationship between DNA sequence and shape, we fused them to the GHTNet. Compared to DNA shape only, the performance of the fused model had significantly improved predictive power (Figure 4B). This result suggests that fusion DNA shape can better understand tissue-specific TF-DNA binding. To further study the importance of DNA structure, DNA shapes were divided into three categories for testing: intra-base, inter-base, and MWG+EP. The importance of each category of DNA shape was analyzed via the strategy that inputs them separately. The average contributions of the three categories of DNA shapes were different. Specifically, the contributions of intra-base, inter-base, and MGW+EP accounted for 36.4%, 37.9%, and 25.6%, respectively (Figure 4A).

We further investigated the importance of each DNA shape in inter-base and intra-base categories via the leave-one-out strategy. The results show that the contribution of each DNA shape was also different. For 12 kinds of shapes in these two categories, Roll, and Buckle was the most important, contributing 25.56% and 37.54% of each type (Figure 4A). Notably, two DNA shapes HelT and Rise negatively contributed to predicting TF-DNA binding. After removing these two shapes, the AUROC increased by 0.0021 and 0.0017, respectively. To investigate the importance of DNA sequence and shape in different tissues, we analyzed the results of three inputs, i.e., DNA sequence, DNA shape, and DNA sequence+shape. The results demonstrate significant differences in the importance of DNA sequence and shape in different tissues. For example, in CTCF and POLR2A, the DNA sequence is quite crucial in the ovary body of pancreas tissues, while less critical in vagina and Peyer’s patch tissues, respectively. DNA shape is essential in gastrocnemius medialis and body of pancreas tissues, while less vital in spleen and thyroid gland tissues, respectively (Figure 4C). This result suggests that DNA sequence and shape have different roles in tissue-specific TF-DNA binding. Overall, the above experiments demonstrate that DNA sequence and shape are remarkable predictors of TF-DNA binding specificity and are crucial factors in tissue-specific TF-DNA binding.

#### 3.2.2. Combining Epigenomics Can Better Understand Tissue-Specific TF-DNA Binding

Epigenetic modifications are essential for regulating gene expression and affecting individual traits without altering the DNA sequence. The expression levels of epigenetic changes vary widely across different tissues. It is also a crucial factor affecting the tissue-specific TF-DNA binding. Thus, we conducted many experiments to analyze DNA sequences, their corresponding HM patterns, and chromatin accessibility.

**Figure 5:**
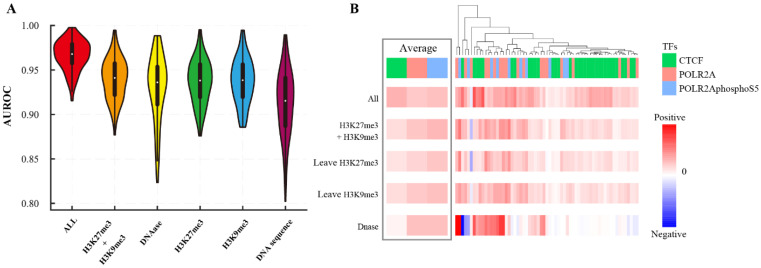
Importance analysis of epigenomics. (**A**) The AUROC of different inputs based on the 86 human ChIP-seq datasets. (**B**) The contribution of different chromatin features combination in predicting CTCF, POLR2A, and POLR2AphosphoS5 in different tissues, where each column represents a dataset.

To evaluate the epigenetics in TF-DNA binding recognition, we applied the GHTNet to three inputs: HM patterns, DNase, and epigenomics combination. Experiments show that only these epigenomics can identify gene transcription binding and that DNase is more important than HM patterns (Supplementary Figure S4). This result may be caused by the high correlation between TF-DNA binding and chromatin accessibility. We also observed that the performance of the same TF varies significantly in different tissues. For example, CTCF was highest in the kidney and lowest in the upper lobe of the left lung, AUROC with a difference of 0.27. To assess tissue-specific TF-DNA binding, we conducted a cross-tissue transfer study. The results demonstrate that epigenetics have strong tissue specificity, and cross-tissue transferred models almost lost their predictive power. The best predictor of TF-DNA binding specificity is DNA sequence, followed by epigenetics and DNA shape (Supplementary Figure S5). To explore the relationship between DNA sequence and epigenomics, we fused them into the GHTNet. The results show that the performance significantly improved when the DNA sequence was combined with epigenetics (Figure 5A), indicating that tissue-specific TF-DNA binding mechanisms can be better understood by comprehensively analyzing these features.

Furthermore, to analyze the importance of each of the epigenetics, we compared the performance differences between models that include them. For clear presentation, the results of three TFs were clustered based on the Pearson correlation, including CTCF, POLR2A, and POLR2AphosphoS5 (Figure 5B). The results show that H3K27me3 and H3K9me3 have similar contributions. Notably, for POLR2A in transverse colon tissue, both HM patterns negatively affected the recognition of its binding. In addition, we found that the contribution of DNase varies widely in different tissues, which suggests that DNase has strong tissue specificity (Figure 5B). These results demonstrate the importance of epigenomics for explaining tissue-specific TF-DNA binding.

### 3.3. Mouse and Human TFs Exhibit Similarities in Tissue-Specific Binding

**Figure 6:**
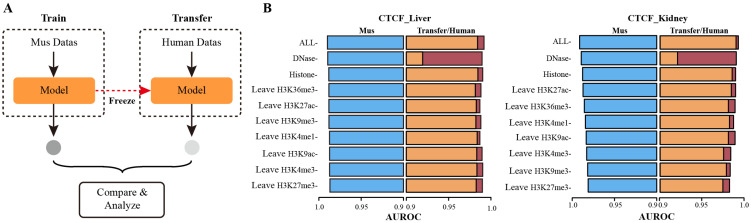
Results of cross-species experiments. (**A**) General flow of cross-species experiments. (**B**) The AUROC comparison between the GHTNet based on mouse and human datasets. The blue box indicates that the model was trained and tested with mouse data, the orange box indicates that the trained with mouse data and tested with human data, and the red box indicates that the trained and tested with human data.

A large amount of data from existing large-scale high-throughput sequencing technologies reveals the mystery of TF-DNA binding specificity. However, collecting all chromatin features from different tissues remains challenging because obtaining samples in some tissues is complex. Since the intrinsic DNA sequence preferences of TFs are usually highly conserved cross-species. Therefore, we hypothesized that the tissue-specific binding mechanism of human TFs could be roughly elucidated by analyzing data from mice. To evaluate this hypothesis, we adopted a transfer methodology, in which the GHTNet was first trained for mice and then transferred to predict the human genome (Figure 6A). This study collected three features: DNA sequence, HM patterns, and DNase. In the following, mouse samples were defined as the experimental group, and human samples were defined as the control group.

Firstly, the GHTNet was applied in the experimental group, resulting in high performance for mice, i.e., the AUROC of CTCF for liver and kidney were 0.9899 and 0.9905, respectively. This result demonstrates the generalizability of the GHTNet, even in different genomes. To investigate the mechanism of TF-DNA binding between species, we transferred the models trained in the experimental group to the control group and compared the results with those of the control group. The experiments show strong similarities in TF-DNA binding features between humans and mice (Supplementary Table S4), perhaps due to the conserved nature of the evolutionary process [34]. For instance, for seven HM patterns of CTCF in liver and kidney tissues, TFBSs in the control group were well predicted after the model transfer with AUROC decreased by less than 0.01. However, DNase shows high specificity between different species. Although the performance significantly reduced after the model was transferred, AUROC was more than 0.9 and remained at a high level (Figure 6B). The above results indicate a high similarity for the TFs binding features in humans and ice.

### 3.4. Downstream Applications

#### 3.4.1. GHTNet Can Be Applied to Identify Important Gene Regions and Motifs

**Figure 7:**
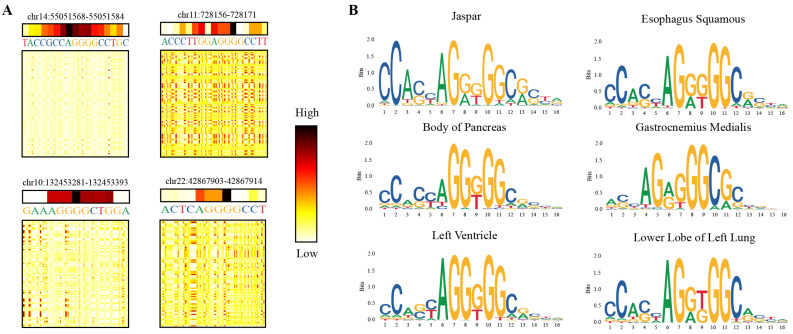
GHTNet identifies important gene regions and motifs. (**A**) Attention scores of four examples of ChIP-seq validated CTCF binding sites in the body of pancreas tissue and its correspondent attention map. The darker the color, the more important the model considers the area. (**B**) The motifs of CTCF in different tissues learned by the GHTNet.

To verify the interpretability of the GHTNet, we utilized a range of methods to interpret the model and visualize the critical regions. Firstly, we visualized the attention map for each attention head and the essential gene regions that influence model decisions. Specifically, attention maps were averaged to obtain attention scores for each position (Supplementary Figure S6). The higher the score, the more vital the gene region. As shown in Figure 7A, the attention map and the attention scores focused on the TF-DNA binding regions to facilitate the model’s decision-making. However, there are still many regions that models consider necessary whose functions are not yet known.

Then, a similar strategy of DeepBind was used for motif identification. It mainly contained three steps: (i) Motif detector selection. Potential motif detectors are selected in the convolution kernels by global max pooling. (ii) Potential TF-DNA binding region identification. In the positive dataset, the maximum activation value of the current motif detector is *l*. For each sequence, the position of the maximum activation value greater than 0.7*l* was selected as the potential binding site. (iii) Motif similarity comparison. To assess the similarity between the motifs identified by the GHTNet and the validated motifs, TOMTOM [37] was utilized as the motif compare tool to evaluate the statistical significance using the JASPAR database. *e*-value, *p*-value, and *q*-value were applied as similarity evaluation metrics. As shown in Figure 7B, the CTCT motifs in different tissues extracted by convolution kernel are similar to those in the JASPAR database but with subtle differences (for more results see Supplementary Figure S11). We suggest that this difference is likely due to tissue-specific TF-DNA binding and possible model error. A total of 91 motifs were identified, 78 motifs were matched to the JASPAR databases, and the other 14 were undocumented (Supplementary Figure S7). To conclude, the GHTNet has excellent interpretability, and the critical biological regions it captures allow us to understand its decision-making process and reveal the mystery of TF-DNA binding.

#### 3.4.2. Significant Links between Transcription Factors, Chromatin Features, and Disease

**Figure 8:**
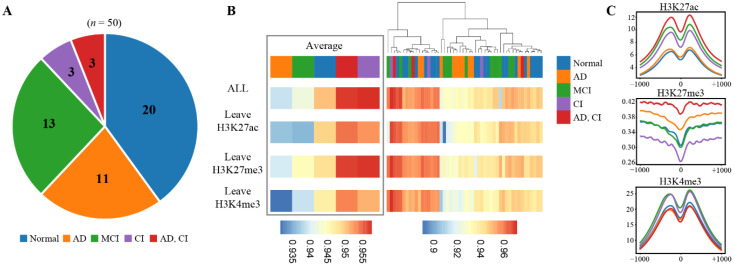
Analysis of disease effects on TF-DNA binding from HM patterns aspect. (**A**) The number of samples per experimental group. (**B**) Heatmap shows the AUROC of 50 samples using different chromatin features, where each column represents a dataset. Those columns were organized by hierarchical clustering, and the categories in each dataset were showcased at the right. (**C**) Average expression levels of H3K27ac, H3K27me3, and H3K4me3 at each position 1kb upstream and downstream from binding sites for CTCF in five experimental groups.

To investigate neurodegenerative diseases, we analyzed AD46 tissue to study diseases such as Alzheimer’s disease (AD) and mild cognitive impairment (MCI). Previous studies demonstrated that neurodegenerative diseases are associated with some TFs, among which CTCF is closely related to cognitive disorders. We collected 50 CTCF samples and their three corresponding HM patterns (H3K27ac, H3K27me3, and H3K4me3) from AD46 tissues. It can be divided into five groups: (i) 20 samples without disease; (ii) 13 samples with MCI; (iii) 11 samples with AD; (vi) three with cognitive impairment (CI); and (v) three with both AD and CI (Figure 8A). 

First, the GHTNet was used to analyze the overall importance of these HM patterns and then adopted the leave-one-out strategy to analyze three HM patterns separately. We found significant differences between samples through the above experiments, while three HM patterns showed equal importance for CTCF binding in a single sample. Although no apparent clustering was demonstrated for these five types of samples, on average the importance of these HM patterns was lower in AD and MCI patients than in the normal group. However, for CI—as well as both AD and CI patients— these HM patterns were more important than the normal group (Figure 8B). We further analyzed the contribution of each HM pattern by the leave-one-out strategy and found variability between samples with no significant clustering. On average, the contribution of each HM pattern was relatively similar in the normal group. However, it differed significantly in the diseased group. For the MCI group, the HM patterns were most significant (Supplementary Figure S8). We randomly sampled each class of samples to construct five datasets of equal size to validate results after repeating the above experiments (Supplementary Figure S9). Recent research has suggested that HM patterns are associated with AD [39]. 

Therefore, the mean expression of three HMs at ±1000 bp from the Chip-seq peak point was visualized to study the relationship between diseases and HM patterns. Meanwhile, the HMs for negative samples were generated for comparison (Supplementary Figure S10). The results show significant differences among the five disease samples compared with the negative samples (Figure 8C). For H3K27ac, the lowest expression level was observed in the normal group and the highest was in the AD group. For H3K27me3, the expression levels of the AD, CI, and AD groups were higher than the normal group, and the MCI and CI groups were lower.

In contrast to H3K27me3, the expression level of the MCI and CI groups was higher than the normal group for H3K4me3, and the expression levels of AD, MCI, and AD groups were lower. Nevertheless, the general trends for each class of samples are similar (Figure 8C), reflecting a close association between disease and HMs expression. The results demonstrate a significant link between TF-DNA binding, epigenomics, and disease; altering them may lead to disease development.

## 4. Discussion

In this work, we developed the GHTNet, a gene transcription binding prediction model based on transformer, which utilized multiple chromatin features to predict TF-DNA binding. Using the GHTNet, we analyzed the relationship between tissue-specific TF-DNA binding and different chromatin features, including 86 samples of 20 TFs in 34 tissues. The important TF-DNA binding features that affect the tissue-specific TF-DNA binding might help explain the differential expression of genes in human tissues and provide clues for diagnosing diseases, developing therapeutic targets, and elucidating disease etiologies. We mainly studied diseases associated with AD46 tissue and revealed the impact of disease occurrence on TF-DNA binding.

The GHTNet used wor2vec, attention mechanism, and CNN to achieve the TF-DNA binding specificity prediction based on multiple factors. We demonstrate that using k-mer for one-hot encoding DNA sequences, performing distributed representation, and incorporating a convolutional network into an FFN in the self-attention module could significantly improve model performance. Using the GHTNet, we separately analyzed DNA shape and epigenomics and found that they both help explain tissue-specific TF-DNA binding. Further, cross-species experiments were conducted to investigate the interspecies specificity. The results show that human and mouse TF-DNA binding mechanisms are very similar, and thus providing new research ideas in a setting with limited data. In addition, the GHTNet can identify essential regions in the genome, and analyzing these crucial regions can provide critical biological insights. In summary, the GHTNet advanced the state of the art in TFBSs prediction, solved the shortcomings of previous studies that could not comprehensively consider local dependence and global dependence, and effectively realized the fusion of various chromatin features. Based on this, we performed many analyses, which provide a solid foundation for elucidating tissue-specific TF-DNA binding mechanisms and understanding the tremendous amount of biological information in the genome sequence. 

The proposed method can be extended in several ways. In this work, we focused on studying different types of chromatin features separately due to limited computing resources. However, their integrated analysis can better understand the tissue-specific TF-DNA binding mechanism. This limitation can be addressed by applying light models, such as ALBERT [40]. The GHTNet can be flexibly extended to identify disease-related genetic variants and prioritize the variant with tissue-specific function. By using an advanced model such as Xlnet [41], we can more thoroughly analyze the genome language and better understand the impact of SNPs. Through the attention mechanism, we can infer regulatory elements and their interactions [6,42]. Due to the DNA 3D spatial structure, distal DNA elements can affect TF-DNA binding [43]. Therefore, using 3D genome information (Hi-C) [44], combined with the graph convolutional neural network (GCN) [45], can lead to better understanding of TF-DNA binding. Advanced models and comprehensive data can provide a complete understanding of gene transcription binding.

## Figures and Tables

**Table 1:** Average performance comparison between GHTNet and competing methods.

Model	AUROC	*p*-Value	Acc	*p*-Value	AUPRC	*p*-Value
GHTNet	0.9667		0.9279		0.9718	
GHTNet-DNA	0.9130	6.85 × 10^−25^	0.8582	2.34 × 10^−24^	0.9235	1.93 × 10^−20^
DeepSEA	0.9080	7.10 × 10^−25^	0.8450	2.04 × 10^−27^	0.9184	1.14 × 10^−21^
CNN_Zeng	0.9086	1.62 × 10^−23^	0.8477	1.30 × 10^−25^	0.9190	6.38 × 10^−20^
DanQ	0.9121	5.16 × 10^−24^	0.8487	3.80 × 10^−25^	0.9215	1.69 × 10^−20^
DLBSS	0.9079	2.20 × 10^−23^	0.8430	2.14 × 10^−27^	0.9164	5.78 × 10^−20^
FactorNet	0.9193	1.45 × 10^−19^	0.8641	1.55 × 10^−19^	0.9309	1.62 × 10^−15^

## Data Availability

The source code of the GHTNet can be found on GitHub at https://github.com/ZhangLab312/GHTNet (accessed on 16 July 2022).

## Supplementary Materials

The following supporting information can be downloaded at: https://www.mdpi.com/article/10.3390/genes13111952/s1, Figure S1: The Skip-gram model. Predicting the probability of multiple words by inputting one word; Figure S2: The architecture of GHTNet-One feature. When the input has only one type of feature, we modified the model to retain only half of GHTNet and its parameters were set in the similar way as the original; Figure S3: Performance of GHTNet and GHTNet-DNA in comparison with five baseline models across three evaluation metrics on 86 human datasets; Figure S4: Importance analysis of two histone modifications and DNase across three evaluation metrics on 86 human datasets; Figure S5: Performance comparison of with three different inputs across three evaluation metrics on 86 human datasets; Figure S6: The extraction process and calculation process of attention map and attention score; Figure S7: Motifs similarity comparison. The −log2(*p*-value), −log2(*e*-value), and −log2(*q*-value) derived from TOMTOM. A total of 78 motifs (known motifs) from GHTNet can be matched to the JASPAR or TRANSFAC, and 14 motifs (undocumented motifs) do not have any matches (pie chart); Figure S8: Contribution analysis of three histone modifications across 50 datasets in AD46 tissue; Figure S9: Contribution analysis of three histone modifications was analyzed by constructing five datasets of equal size through random sampling of each class of samples; Figure S10: Average expression levels of H3K27ac, H3K27me3, and H3K4me3 of negative samples for CTCF in five groups; Figure S11: Comparison of the similarity between CTCF motifs identified by GHTNet and validated motifs in different tissues; Figure S12: The encoding process of DNA sequences; Figure S13: Performance comparison between GHTNet and Transformer; Table S1: The datasets we used, which can be divided t into three categories according to the research content; Table S2: Performance comparison of different TFs on different tissues; Table S3: Mean performance of GHTNet (with *k*-mer = 2, 3, 4, 5, 6) on 86 ChIP-seq human TF datasets; Table S4: Results of cross-species studies, suggesting a high degree of conservation between humans and mice; Table S5. The architecture of our proposed model GHTNet. References [46,47,48,49,50,51] are cited in the supplementary materials.
