# Supplementary material for: Uncovering the Relationship between Tissue-Specific TF-DNA Binding and Chromatin Features through a Transformer-Based Model
Source: Genes (Basel). 2022 Oct 26;13(11):1952. doi: 10.3390/genes13111952 (PMC9690320; doi:10.3390/genes13111952)
Supplement: Supplementary file 1 [file genes-13-01952-s001.zip › Figure S11.pdf]

S11 Fig

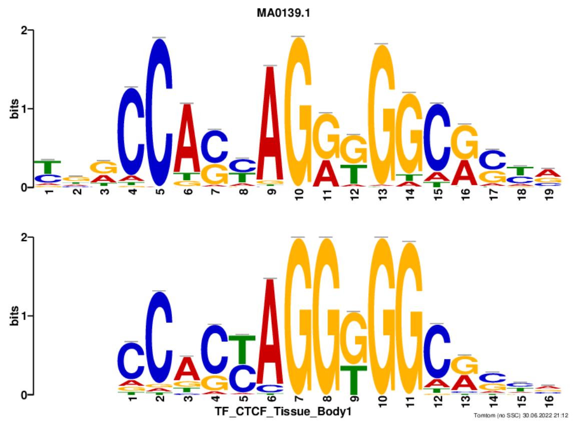

TF	CTCF
Tissue	Body of pancreas
P-value	2.69e-18
E-value	5.25e-15
q-value	1.05e-14

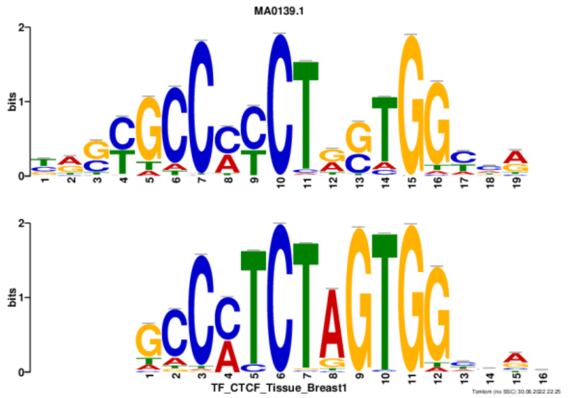

TF	CTCF
Tissue	Breast epithelium
P-value	1.53e-08
E-value	2.99e-05
q-value	5.98e-05

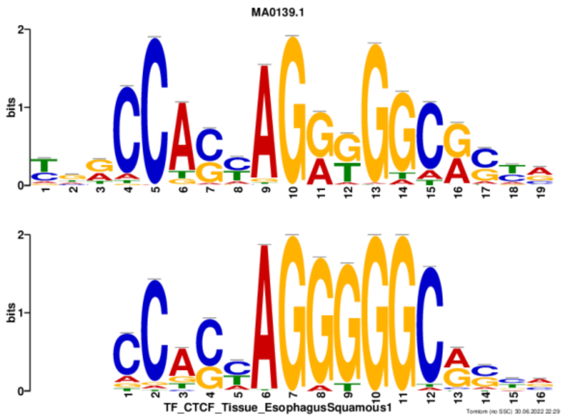

TF	CTCF
Tissue	Esophagus squamous
P-value	3.06e-16
E-value	5.99e-13
q-value	1.19e-12

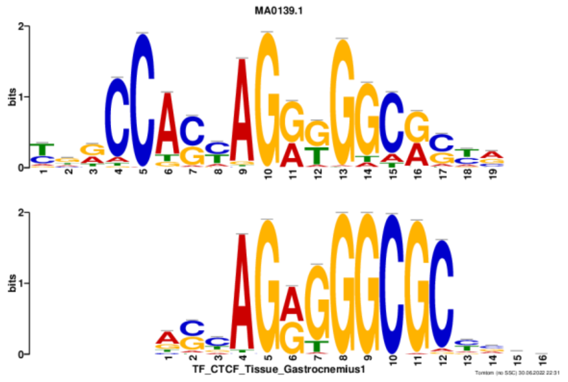

TF	CTCF
Tissue	Gastrocnemius
P-value	1.41e-08
E-value	2.76e-05
q-value	2.76e-05

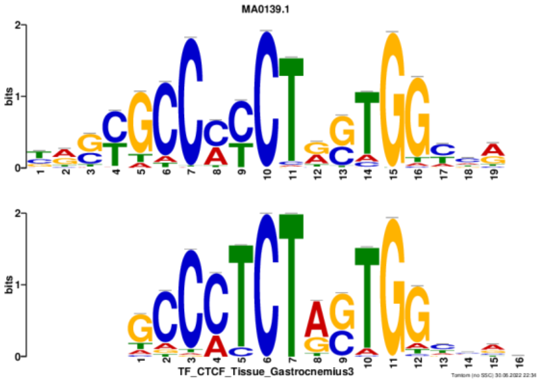

TF	CTCF
Tissue	Gastrocnemius
P-value	1.13e-08
E-value	2.22e-05
q-value	4.43e-05

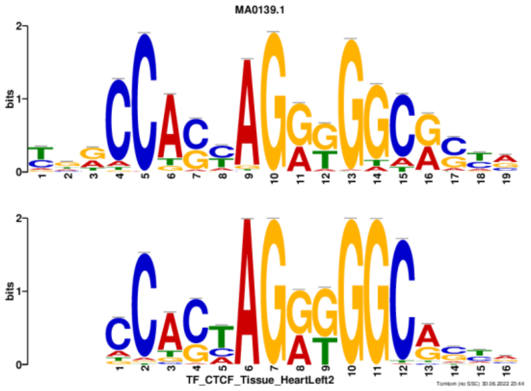

TF	CTCF
Tissue	Heart left ventricle
P-value	9.15e-15
E-value	1.79e-11
q-value	1.79e-11

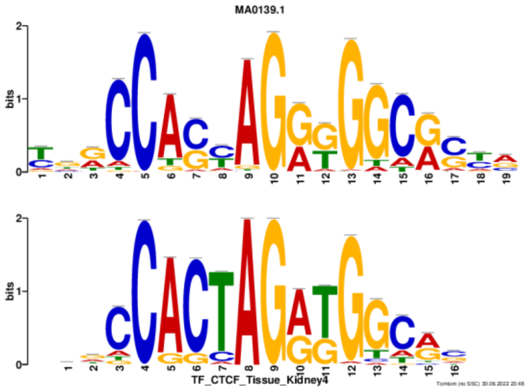

TF	CTCF
Tissue	Kidney
P-value	4.90e-11
E-value	9.58e-08
q-value	9.58e-08

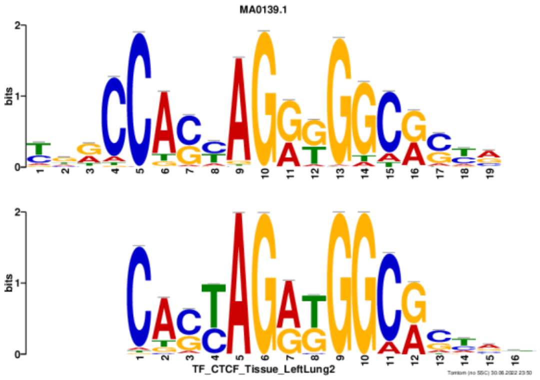

TF	CTCF
Tissue	Left lung
P-value	1.96e-10
E-value	3.84e-07
q-value	3.84e-07

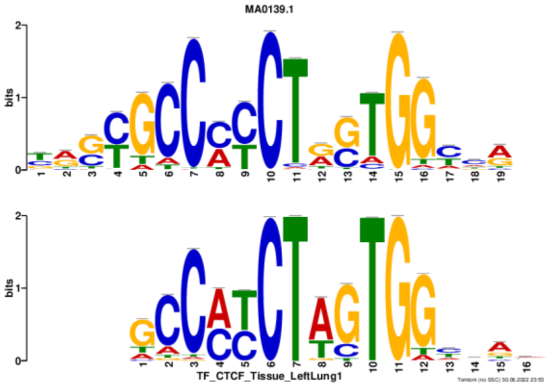

TF	CTCF
Tissue	Left lung
P-value	6.38e-09
E-value	1.25e-05
q-value	2.50e-05

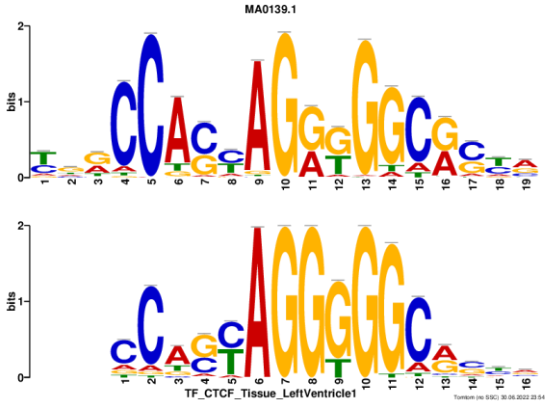

TF	CTCF
Tissue	Left ventricle myocardium
P-value	7.55e-16
E-value	1.48e-12
q-value	2.94e-12

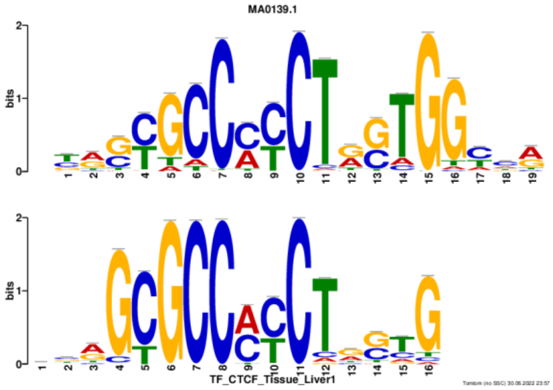

TF	CTCF
Tissue	Liver
P-value	1.80e-11
E-value	3.51e-08
q-value	4.21e-08

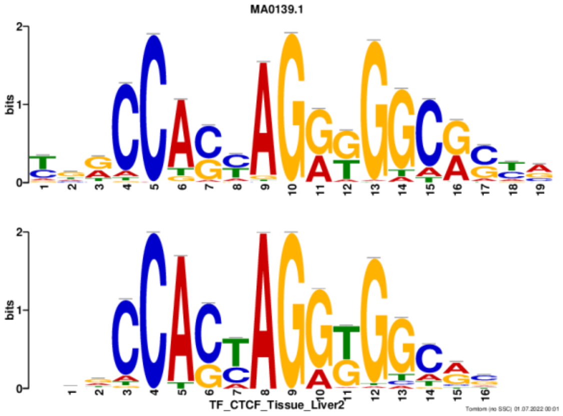

TF	CTCF
Tissue	Liver
P-value	7.45e-16
E-value	1.46e-12
q-value	1.56e-12

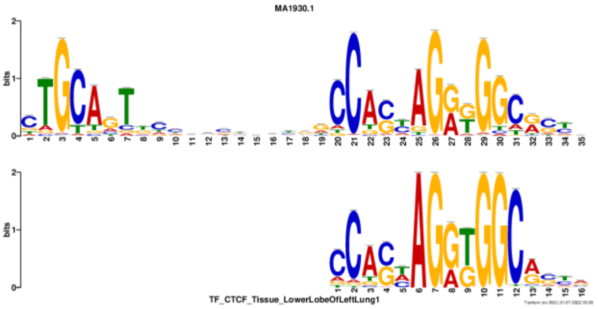

TF	CTCF
Tissue	Lower lobe of left lung
P-value	8.14e-16
E-value	1.59e-12
q-value	3.18e-12

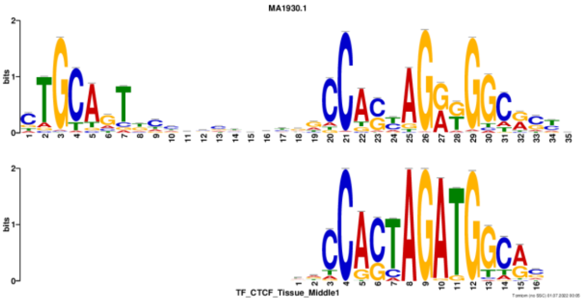

TF	CTCF
Tissue	Middle frontal area 46
P-value	1.06e-07
E-value	2.07e-04
q-value	3.18e-04

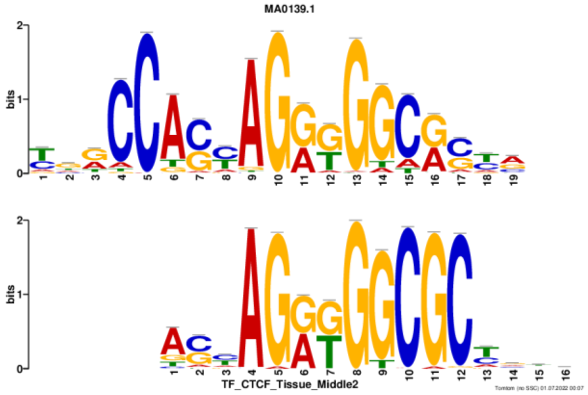

TF	CTCF
Tissue	Middle frontal area 46
P-value	4.57e-09
E-value	8.94e-06
q-value	1.78e-05

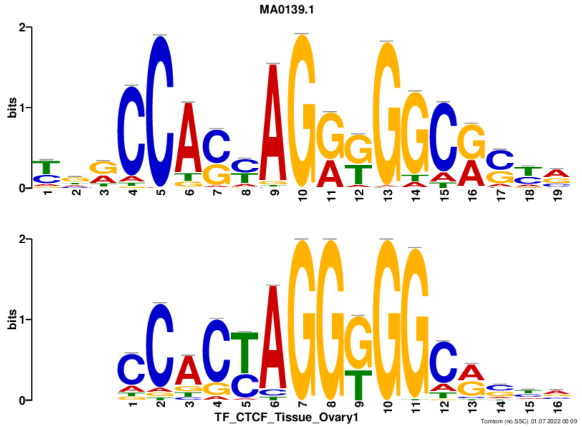

TF	CTCF
Tissue	Ovary
P-value	8.75e-16
E-value	1.71e-12
q-value	3.42e-12

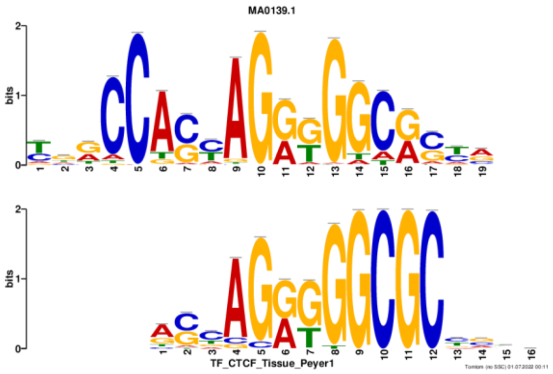

TF	CTCF
Tissue	Peyer's patch testis
P-value	2.98e-09
E-value	5.82e-06
q-value	8.34e-06

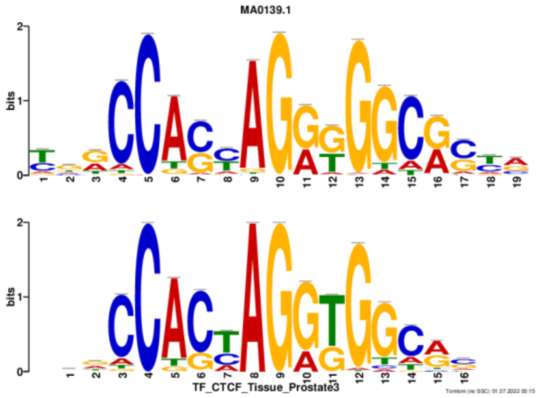

TF	CTCF
Tissue	Prostate gland
P-value	1.95e-13
E-value	3.82e-10
q-value	7.20e-10

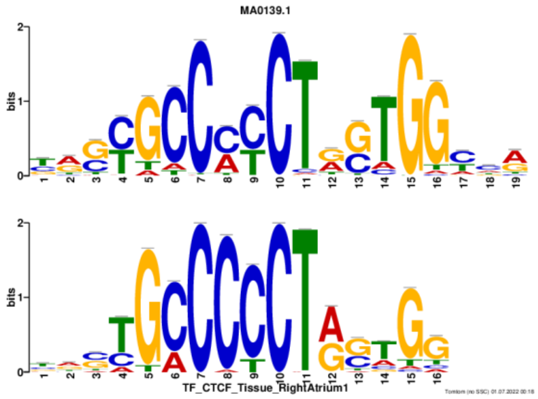

TF	CTCF
Tissue	Right atrium auricular region
P-value	4.44e-13
E-value	8.68e-10
q-value	1.73e-09

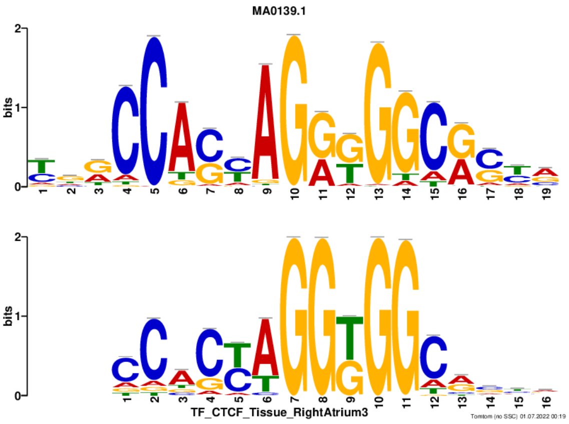

TF	CTCF
Tissue	Right atrium auricular region
P-value	5.68e-16
E-value	1.11e-12
q-value	2.22e-12

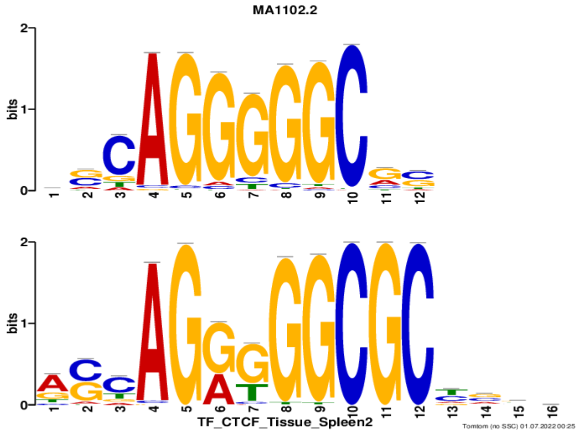

TF	CTCF
Tissue	Spleen
P-value	2.83e-09
E-value	5.54e-06
q-value	1.09e-05

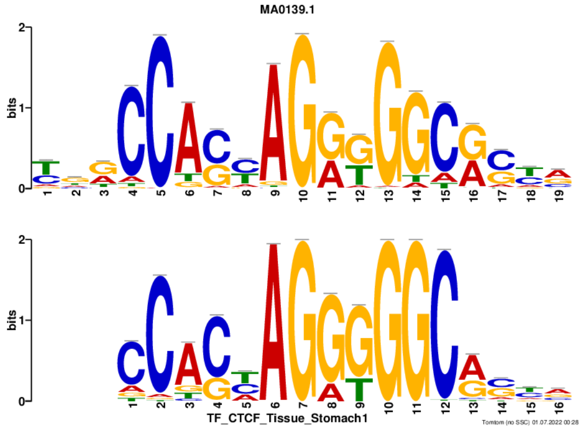

TF	CTCF
Tissue	Stomach
P-value	5.31e-16
E-value	1.04e-12
q-value	2.07e-12

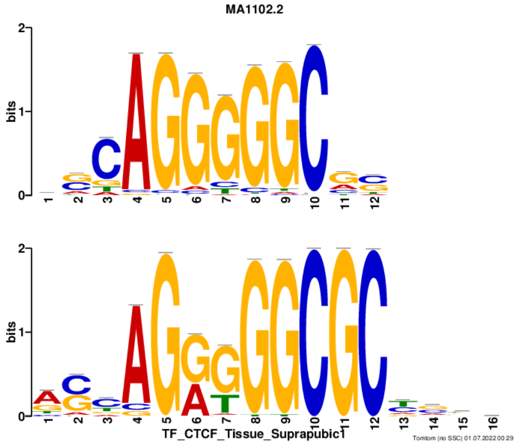

TF	CTCF
Tissue	Suprapubic skin
P-value	2.54e-09
E-value	4.96e-06
q-value	9.79e-06

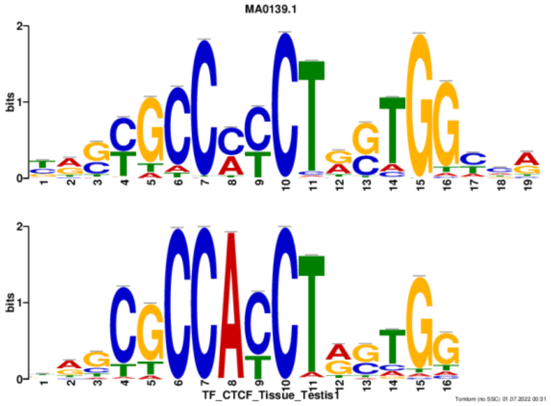

TF	CTCF
Tissue	Testis
P-value	1.98e-11
E-value	3.88e-08
q-value	7.73e-08

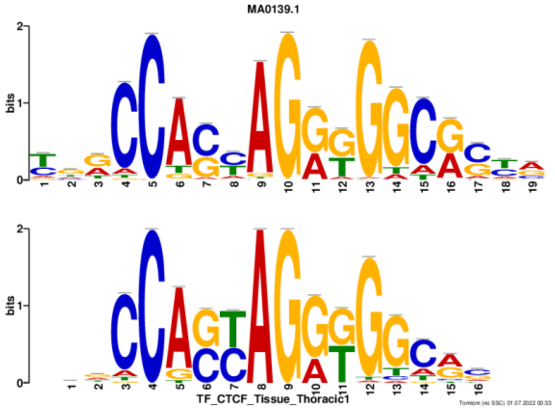

TF	CTCF
Tissue	Thoracic aorta
P-value	1.57e-18
E-value	3.07e-15
q-value	6.09e-15

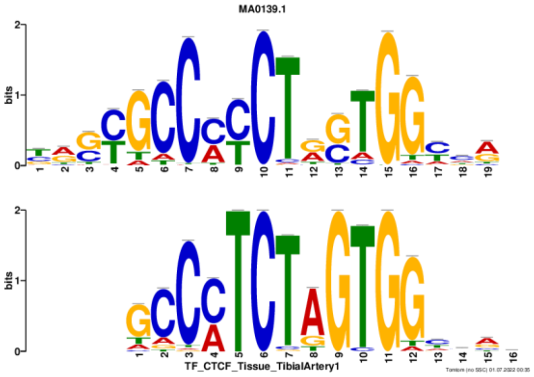

TF	CTCF
Tissue	Tibial artery
P-value	8.23e-09
E-value	1.61e-05
q-value	3.22e-05

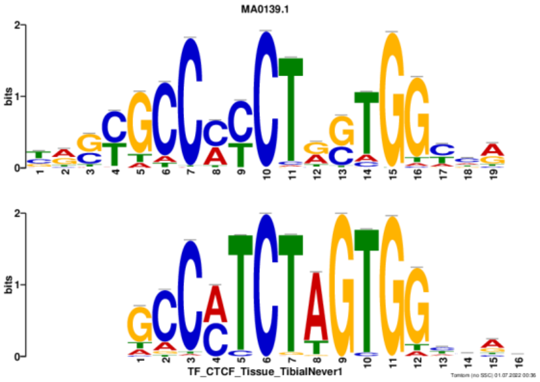

TF	CTCF
Tissue	Tibial nerve
P-value	3.63e-08
E-value	7.09e-05
q-value	7.09e-05

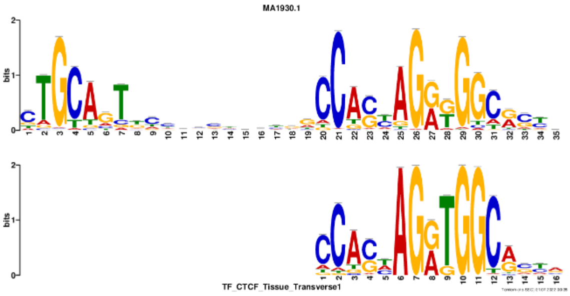

TF	CTCF
Tissue	Transverse colon
P-value	2.78e-13
E-value	5.43e-10
q-value	1.09e-09

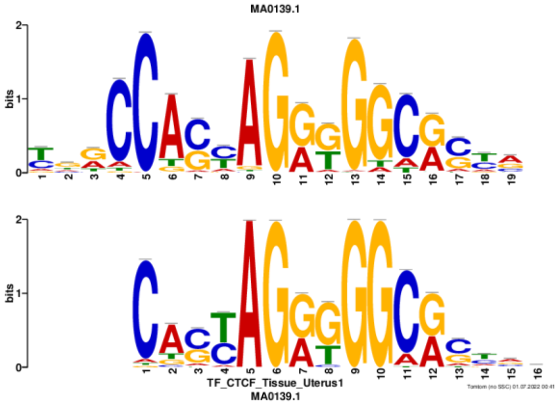

TF	CTCF
Tissue	Upper lobe of left lung
P-value	3.11e-07
E-value	6.09e-04
q-value	1.18e-03

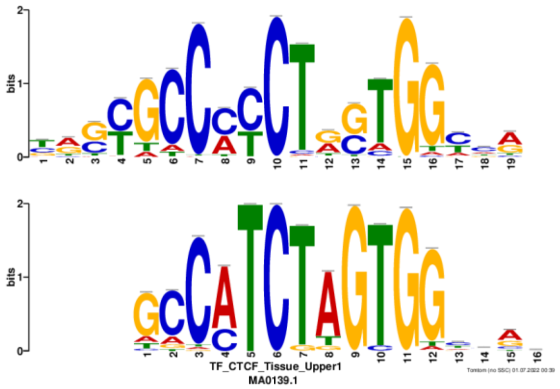

TF	CTCF
Tissue	Uterus
P-value	7.29e-13
E-value	1.43e-09
q-value	2.85e-09

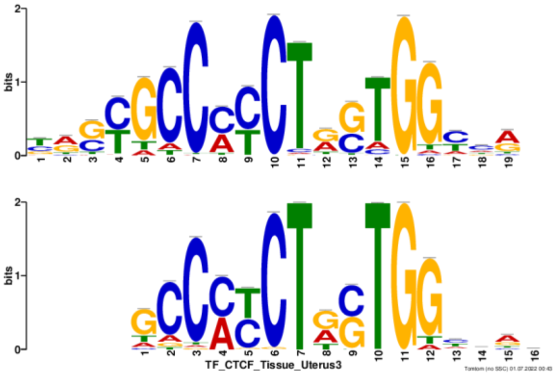

TF	CTCF
Tissue	Uterus
P-value	2.76e-13
E-value	5.39e-10
q-value	1.07e-09

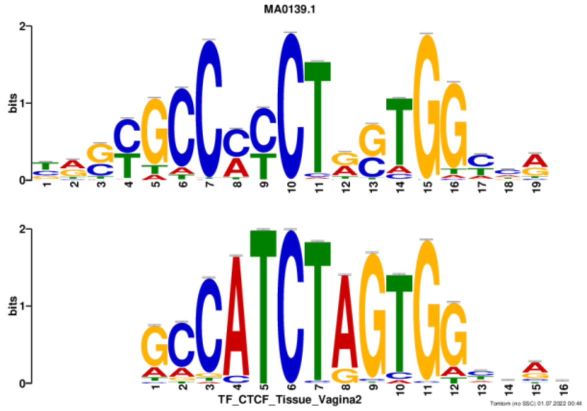

TF	CTCF
Tissue	Vagina
P-value	5.03e-07
E-value	9.85e-05
q-value	1.97e-04

S11 Fig. Comparison of the similarity between CTCF motifs identified by GHTNet and validated motifs in different tissues.
