# Supplementary material for: Uncovering the Relationship between Tissue-Specific TF-DNA Binding and Chromatin Features through a Transformer-Based Model
Source: Genes (Basel). 2022 Oct 26;13(11):1952. doi: 10.3390/genes13111952 (PMC9690320; doi:10.3390/genes13111952)
Supplement: Supplementary file 1 [file genes-13-01952-s001.zip › Supplementary Materials.pdf]

Supplemental information for *Uncovering the relationship between tissue-specific TF-DNA binding and chromatin features through a Transformer-based model*

Yongqing Zhang[1], Yuhang Liu[1], Zixuan Wang[1], Maocheng Wang[1], Shuwen Xiong[1], Guo Huang[2] and Meiqin Gong[3,*]

[1]School of Computer Science, Chengdu University of Information Technology, Chengdu, China;

[2]School of Electronic Information and Artificial Intelligence, Leshan Normal University, Leshan, Chain;

[3]West China Second University Hospital, Sichuan University, Chengdu, China

S1 Text

Acc: The Acc is expressed as the proportion of the model's correct predictions in all samples. The following formula can express it.

$$Acc = \frac{TP + TN}{TP + TN + FP + FN}$$

AUROC: This metric represents the area under the ROC curve, indicating the probability that the model ranks a random positive example higher than a random negative example. The following formula can express it.

$$AUROC = \frac{\sum_{i \in positiveclass} ranki - \frac{M(1+M)}{2}}{M \times N}$$

AUPRC: This metric represents the area under the precision-recall curve and is a better indicator of model performance than ROC-AUC when the data are unbalanced. The following formula can express it.

$$AUPRC = \sum_{k=1}^{n} P(k) \Delta r(k)$$

S2 Text

Encoding module: According to our encoding methods, the DNA sequence is encoded using the word2vec strategy [1], which can be represented as follows:

$$S_m = [o_1, o_2, \dots, o_i, \dots, o_{100}, o_{101}] \quad \text{(S1)}$$

where o_i represents the distributed representation of the i-th segment. To incorporate other four TF-DNA binding features into GHTNet, these features were generated as a feature matrix directly and signified by S_m, S_h, S_d, and S_c. And the dimension of these feature matrix is $n \times m$ and where m represents the number of these four types (i.e. for DNA shape, $m = 14$). These feature matrices can be represented as follows

$$S_m = [m_1, m_2, \dots, m_i, \dots, m_{100}, m_{101}] \quad \text{(S2)}$$

$$S_h = [h_1, h_2, \dots, h_i, \dots, h_{100}, h_{101}] \quad \text{(S3)}$$

$$S_d = [d_1, d_2, \dots, d_i, \dots, d_{100}, d_{101}] \quad \text{(S4)}$$

$$S_c = [c_1, c_2, \dots, c_i, \dots, c_{100}, c_{101}] \quad \text{(S5)}$$

where m_i, h_i, d_i, and c_i represent the Monte-Carlo simulation vector [2], histone ChIP-seq value [3], DNase-seq value [4], and conservation score [5] corresponding to the i-th of segment. Then, these feature matrices were fed into the C-Transformer module to capture long- and short-distance dependence.

Pseudo siamese C-Transformer module: A pseudo siamese network [6] based on Transformer [7] were constructed, due to the various importance of different inputs. The input of this pseudo siamese network could be (i) [S_o] and [S_m, S_h, S_d, S_c], (ii) [S_o] and [S_m], (iii) [S_o] and [S_h], and (iv) [S_o] and [S_h, S_d], where [·,·] represents concatenating them in the channel dimension (i.e. [S_m, S_h, S_d, S_c] means that concatenate four $n \times 14$, $n \times 2$, $n \times 1$, and $n \times 1$ matrices into $n \times 18$ dimension). Each input was first added with the position information P, which can solve the problem of polysemy

$$\tilde{X} = X + P \quad \text{(S6)}$$

here, P represents positional encoding, as the model parameter, which is a $n \times 1$ weight matrix, initialized to 0.01. Then $\tilde{X}$ was fed into the multi-head self-attention layer to capture position dependency information. A residual network was used in this layer, and followed by layer normalization. As shown in formula S7:

$$A = LM\left(MultiHead\left(\tilde{X}, W_m^Q, W_m^K, W_m^O\right) + \tilde{X}\right) \quad \text{(S7)}$$

where A represents the output of multi-head self-attention; $MultiHead(\cdot)$ represents the multi-head attention mechanism; $LM(\cdot)$ stands for layer normalization operation. Specifically, multi-head self-attention mechanism can be shown as follows:

$$MultiHead\left(\tilde{X}, W_m^Q, W_m^K, W_m^O\right) = Concat\left(H_1, \ldots, H_i, \ldots, H_h\right) W_m^O \quad \text{(S8)}$$

$$\text{where, } H_i = softmax\left(\frac{\tilde{X}W_{m(i)}^Q\left(\tilde{X}W_{m(i)}^K\right)^T}{\sqrt{d_k}}\right)\tilde{X}W_{m(i)}^K \quad \text{(S9)}$$

where H_i represents the output of the i-th attention head; W_m^Q, W_m^K, W_m^O represent the weight matrix; $Concat(\cdot)$ means concatenation operation. Using $\sqrt{d_k}$ for scaling to prevent the inner product of $\tilde{X}W_i^Q\left(\tilde{X}W_i^K\right)^T$ from being too large, where $d_k = d_{model} / h$. And $h = d_{model} // 2$ indicates the number of attention head, where d_{model} is equal to the input feature dimension.

Next, A was fed into the C-FFN, which added convolution operation to the FFN. C-FFN can be defined as follows:

$$\widetilde{M} = Relu\left(MLP\left(A, W_{m(0)}, b_{m(0)}\right)\right) \quad \text{(S10)}$$

$$C = MaxPool\left(Relu\left(Conv\left(M', W_{c(0)}, b_{c(0)}\right)\right)\right) \quad \text{(S11)}$$

$$M = Relu\left(MLP\left(C, W_{m(1)}, b_{m(1)}\right)\right) \quad \text{(S12)}$$

where M represents the output of C-FFN; $W_{m(0)}$, $W_{c(0)}$, $W_{m(1)}$ are the weight matrices of the corresponding network; $b_{m(0)}$, $b_{c(0)}$, $b_{c(1)}$ are the bias matrices of the corresponding network. Specifically, $W_{c(0)}$ represents convolution filters, each of which is a $l \times \gamma$ matrix with $l = 3$ and γ = dmodel × 2 and can extract low-range features. Followed by max pooling layer that can downsampling to the activation score vectors to reduce the dimension. The shapes of M_0 and M are $[n, d_{model} \times 2]$ and $[n, d_{model}]$, respectively. Finally, a residual layer and layer normalization were used:

$$Z' = LM(M + A) \quad \text{(S3)}$$

where Z' represent the output of one C-Transformer layer. After a forward pass through $L = 2$ layers, we got the final output of this module Z, where L represents the number of C-Transformer layers.

Pseudo siamese CNN module: CNN [8] was used to extracrt gene transcription binding features. For DNA sequence, it can be regarded as motif detector. When given input Z from pervious module, it can be defined as follows:

$$F' = MaxPool\left(Relu\left(Conv\left(Z, W_c^1, b_c^1\right)\right)\right) \qquad (S14)$$

where F' represents the extracted features.In this part, two convolutional layers were used and obtained the final output F. Specifically, F_d and F_o corresponded to the output of DNA sequence and other features in the Siamese network. Finally, we concatenated the output of the siamese network F_d and F_o and fed into a fully connected layer [9]. Formally, the above operations are defined as follows:

$$\hat{Y} = Sigmoid\left(MLP\left(Concat\left(F_d, F_o\right), W_f, b_f\right)\right) \qquad (S15)$$

where $\hat{Y}$ represents the predicted probability of the TF-DNA binding specificity in current sequence, which range from 0 to 1. This part only has one hidden layer with 925 neurons and used the *ReLU* activation function [10]. The final output layer only has two neurons and the Softmax activation function was used to obtain the final output.

The steps for each input sequence is summarized in blow.

Algorithm S1 GHTNet modeling

Input: The DNA sequences containing TFBSs or non-TFBSs.

Output: Prediction value y_j of the current input sequence and all the learned parameters.

1：The DNA sequence is divided into k-mer base segments, which are represented by the feature matrix S_o according to the word list calculated by word2vec. Meanwhile, the DNA shape, histone modification feature, DNase data and conservation score were extracted based on the current DNA sequence and expressed as feature matrices S_m, S_h, S_d, S_c. The above process can be seen in Equations (S1), (S2), (S3), (S4), and (S5).;

2：Initialize all the parameters Θ of neural network;

3：**while** Epoch < MaxEpoch and Eearly stop==False **do**

4： Compute the output of the C-Transformer module according to Equation (S6)-(S13);

5： Using CNN to extract feature. according to Equation (S14);

7： Compute the prediction value y_j of the current input sequence according to Equation (S15);

8： Compute the loss L;

9： Update the parameters Θ;

10： Epoch = Epoch+1

11：**end while**

Main notations used in this paper is summarized in blow.

Notations	Description
S_o, S_m, S_h, S_d, S_c	The feature encoding matrix of DNA sequence, DNA shape, histone modification, chromatin accessibility, and conservation score, respectively.
o_i, m_i, h_i, d_i, c_i	The values of the DNA encoding vector, Monte-Carlo simulation vector, histone modification vector, chromatin accessibility vector, and conservation score vector corresponding to the i-th nucleotide.
P	Position information.
W,b	Weight matrix and bias vector, respectively.
H	Mulit-head attention.
A	The output of the mulit-head attention.
X, $\tilde{X}$	The original feature matrix and the feature matrix with position information.
C	Combined latent feature.
Z', Z	The output of one C-Transformer layer and final output of N C-Transformer layer.
F	Combined latent feature.
y_i, $\hat{y}_i$	Ground-true and predicted probability of i-th input sequence.
Θ	All the parameters in our model.
Θ	All the parameters in our model.

References

1. Mikolov T, Chen K, Corrado G, Dean J. Efficient estimation of word representations in vector space. arXiv preprint arXiv:13013781. 2013.
2. Li J, Sagendorf JM, Chiu T-P, Pasi M, Perez A, Rohs R. Expanding the repertoire of DNA shape features for genome-scale studies of transcription factor binding. Nucleic acids research. 2017;45(22):12877-87.
3. Park PJ. ChIP–seq: advantages and challenges of a maturing technology. Nature reviews genetics. 2009;10(10):669-80.
4. Song L, Crawford GE. DNase-seq: a high-resolution technique for mapping active gene regulatory elements across the genome from mammalian cells. Cold Spring Harbor Protocols. 2010;2010(2):pdb. prot5384.
5. Siepel A, Bejerano G, Pedersen JS, Hinrichs AS, Hou M, Rosenbloom K, et al. Evolutionarily conserved elements in vertebrate, insect, worm, and yeast genomes. Genome research. 2005;15(8):1034-50.
6. Chopra S, Hadsell R, LeCun Y, editors. Learning a similarity metric discriminatively, with application to face verification. 2005 IEEE Computer Society Conference on Computer Vision and Pattern Recognition (CVPR'05); 2005: IEEE.
7. Vaswani A, Shazeer N, Parmar N, Uszkoreit J, Jones L, Gomez AN, et al. Attention is all you need. Advances in neural information processing systems. 2017;30.
8. LeCun Y, Bottou L, Bengio Y, Haffner P. Gradient-based learning applied to document recognition. Proceedings of the IEEE. 1998;86(11):2278-324.

9. Cybenko G. Approximation by superpositions of a sigmoidal function. Mathematics of control, signals and systems. 1989;2(4):303-14.
10. Krizhevsky A, Sutskever I, Hinton GE. Imagenet classification with deep convolutional neural networks. Advances in neural information processing systems. 2012;25.

Legends for Supplementary Tables

Supplementary Table S1. The datasets we used, which can be divided t into three categories according to the research content.

Supplementary Table S2. Performance comparison of different TFs on different tissues.

Supplementary Table S3. Mean performance of GHTNet (with *k*-mer = 2, 3, 4, 5, 6) on 86 ChIP-Seq human TF datasets.

Supplementary Table S4. Results of cross-species studies, suggesting a high degree of conservation between humans and mice.

Supplementary Table S5. Architecture of our proposed model GHTNet.

Legends for Supplementary Figures

Supplementary Figure S1. The Skip-gram model. Predicting the probability of multiple words by inputting one word.

Supplementary Figure S2. The architecture of GHTNet-One feature. When the input has only one type of feature, we modified the model to retain only half of GHTNet and its parameters were set in the similar way as the original.

Supplementary Figure S3. Performance of GHTNet and GHTNet-DNA in comparison with five baseline models across three evaluation metrics on 86 human datasets.

Supplementary Figure S4. Importance analysis of two histone modifications and DNase across three evaluation metrics on 86 human datasets.

Supplementary Figure S5. Performance comparison of with three different inputs across three evaluation metrics on 86 human datasets.

Supplementary Figure S6. The extraction process and calculation process of

attention map and attention score.

Supplementary Figure S7 Motifs similarity comparison. The −log2(P-value), −log2(E-value), and −log2(q-value) derived from TOMTOM. A total of 78 motifs (known motifs) from GHTNet can be matched to the JASPAR or TRANSFAC, and 14 motifs (undocumented motifs) do not have any matches (pie chart).

Supplementary Figure S8. Contribution analysis of three histone modifications across 50 datasets in AD46 tissue.

Supplementary Figure S9. Contribution analysis of three histone modifications was analyzed by constructing five datasets of equal size through random sampling of each class of samples.

Supplementary Figure S10. Average expression levels of H3K27ac, H3K27me3, and H3K4me3 of negative samples for CTCF in five groups.

Supplementary Figure S11. Comparison of the similarity between CTCF motifs identified by GHTNet and validated motifs in different tissues.

Supplementary Figure S12. The encoding process of DNA sequences.

Supplementary Figure S13. Performance comparison between GHTNet and Transformer.
