# Supplementary figures and images for: Uncovering the Relationship between Tissue-Specific TF-DNA Binding and Chromatin Features through a Transformer-Based Model
Source: Genes (Basel). 2022 Oct 26;13(11):1952. doi: 10.3390/genes13111952 (PMC9690320; doi:10.3390/genes13111952)

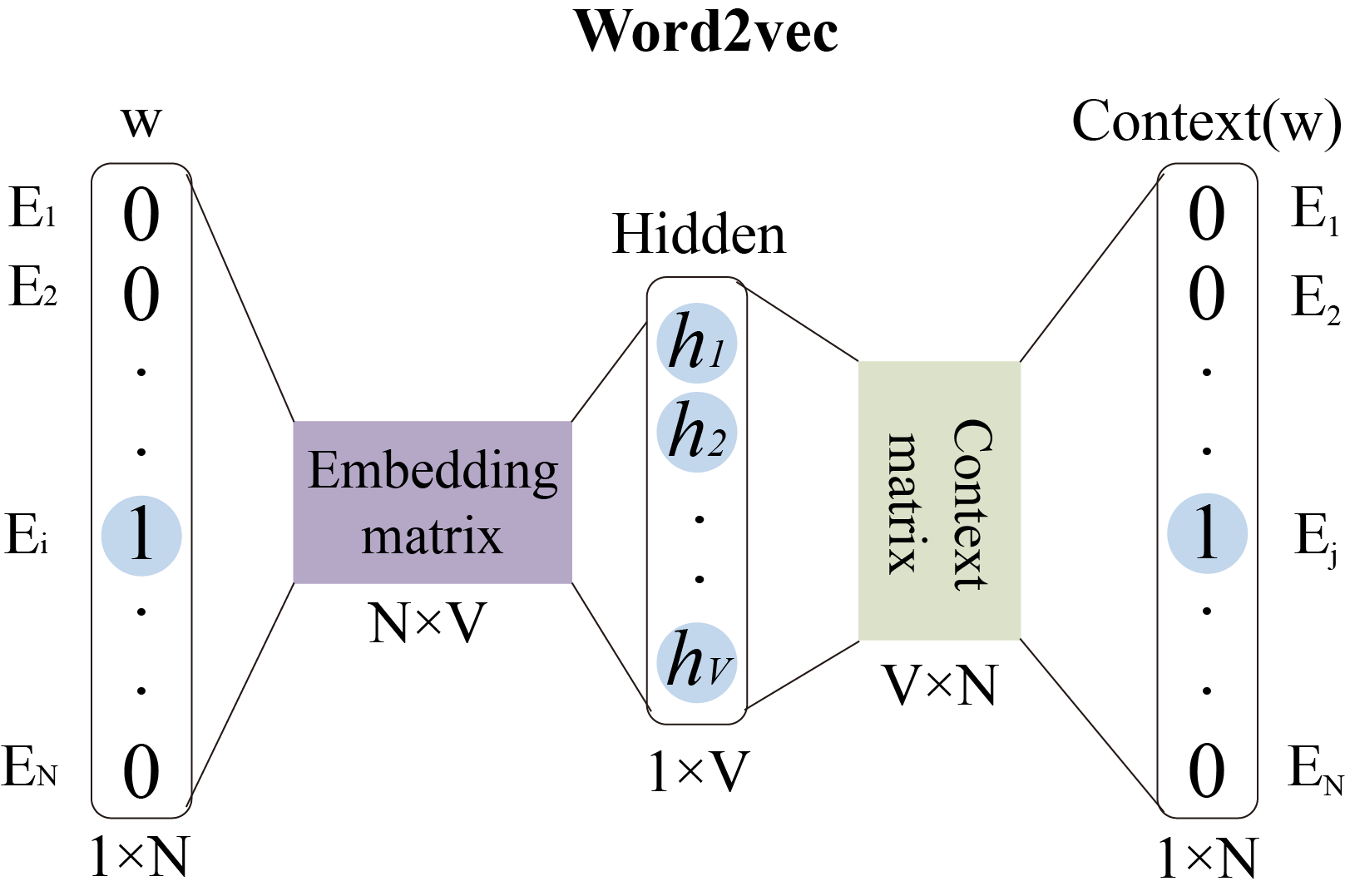

Supplement: Supplementary file 1 [file genes-13-01952-s001.zip › Figure S1.tif]

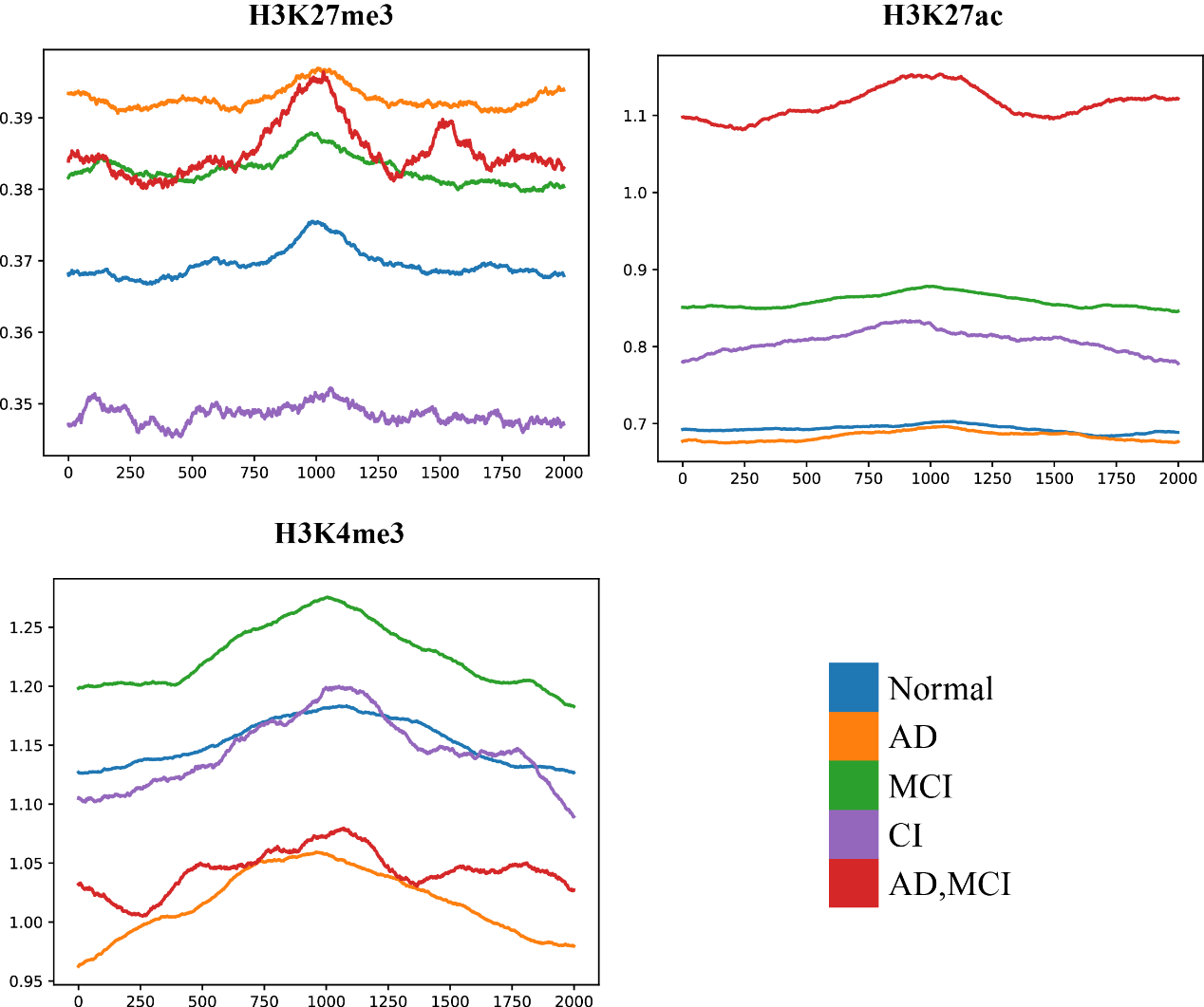

Supplement: Supplementary file 1 [file genes-13-01952-s001.zip › Figure S10.tif]

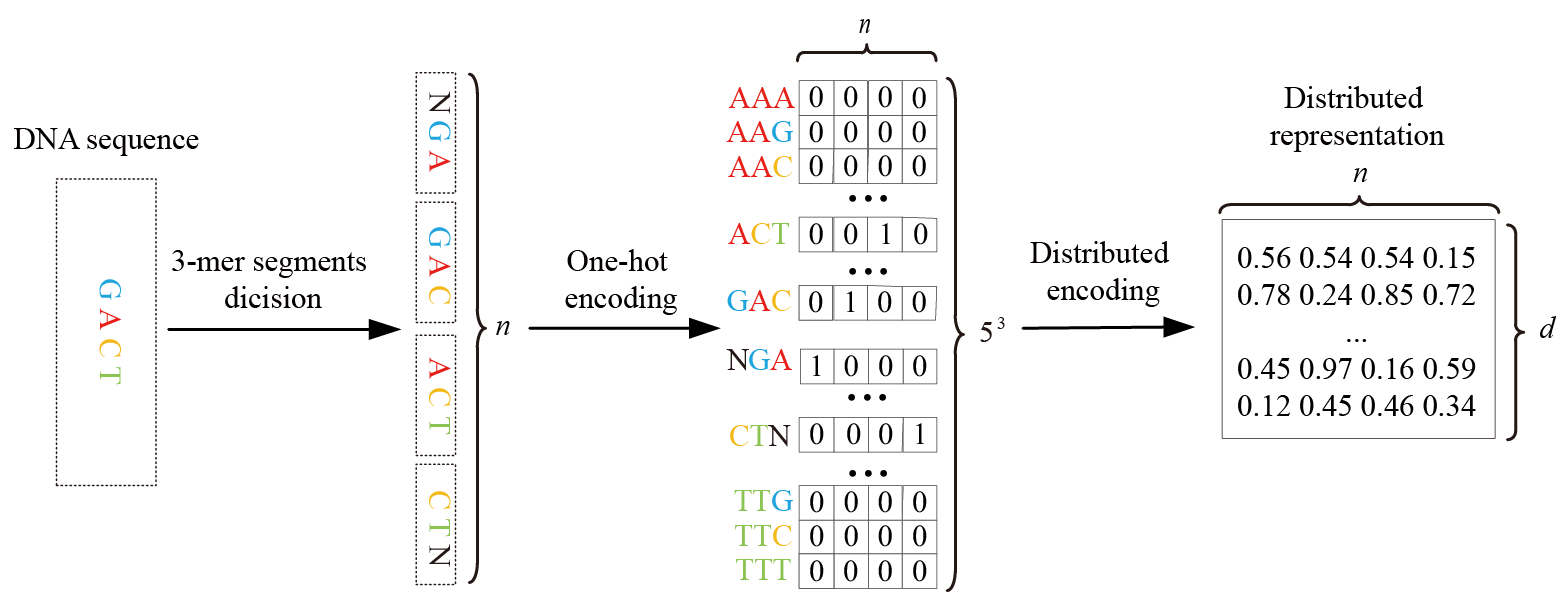

Supplement: Supplementary file 1 [file genes-13-01952-s001.zip › Figure S12.tif]

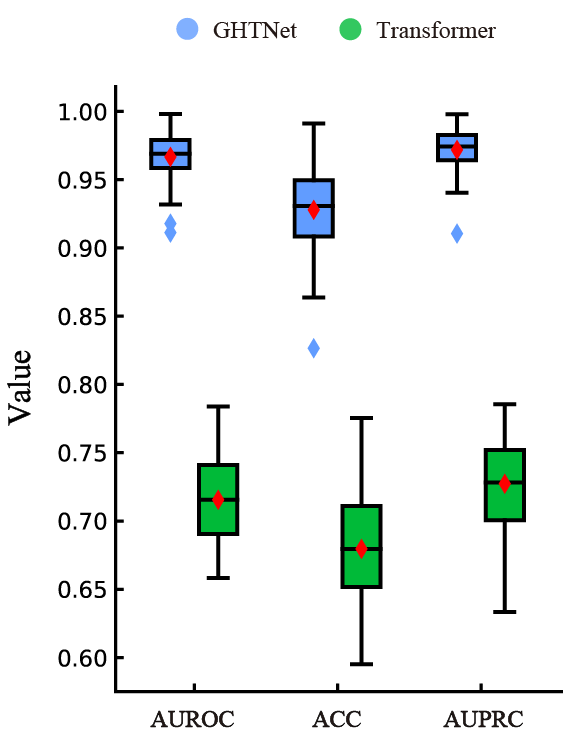

Supplement: Supplementary file 1 [file genes-13-01952-s001.zip › Figure S13.tif]

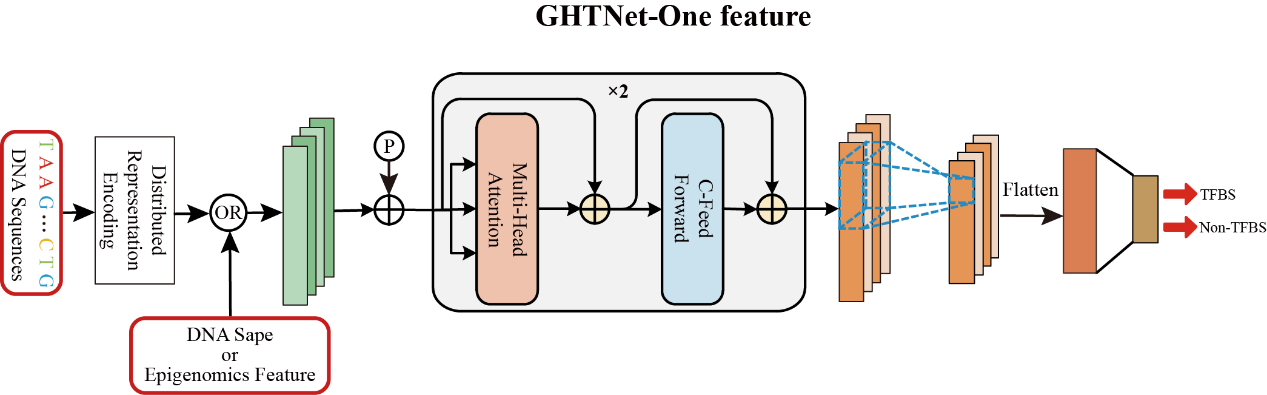

Supplement: Supplementary file 1 [file genes-13-01952-s001.zip › Figure S2.tif]

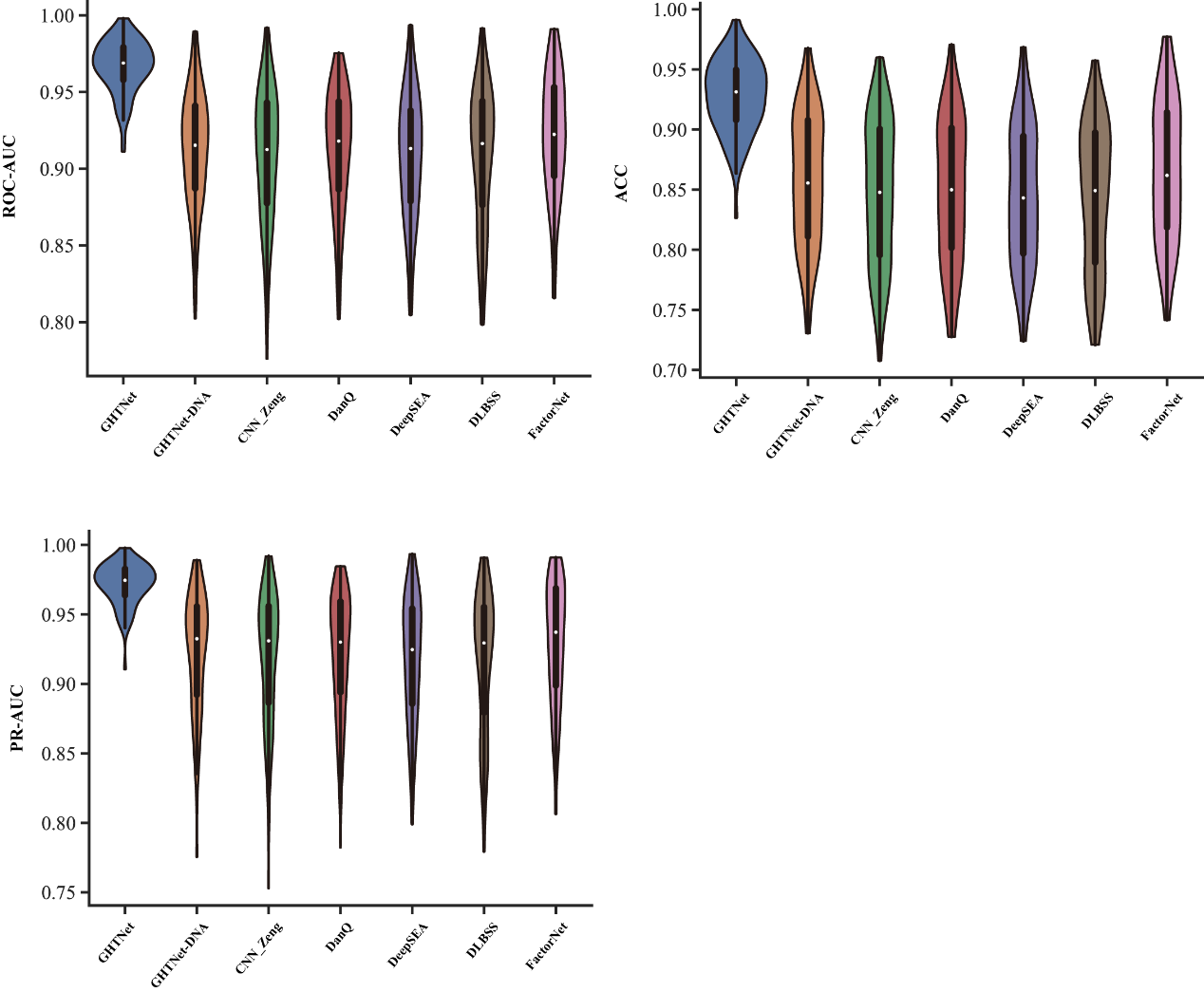

Supplement: Supplementary file 1 [file genes-13-01952-s001.zip › Figure S3.tif]

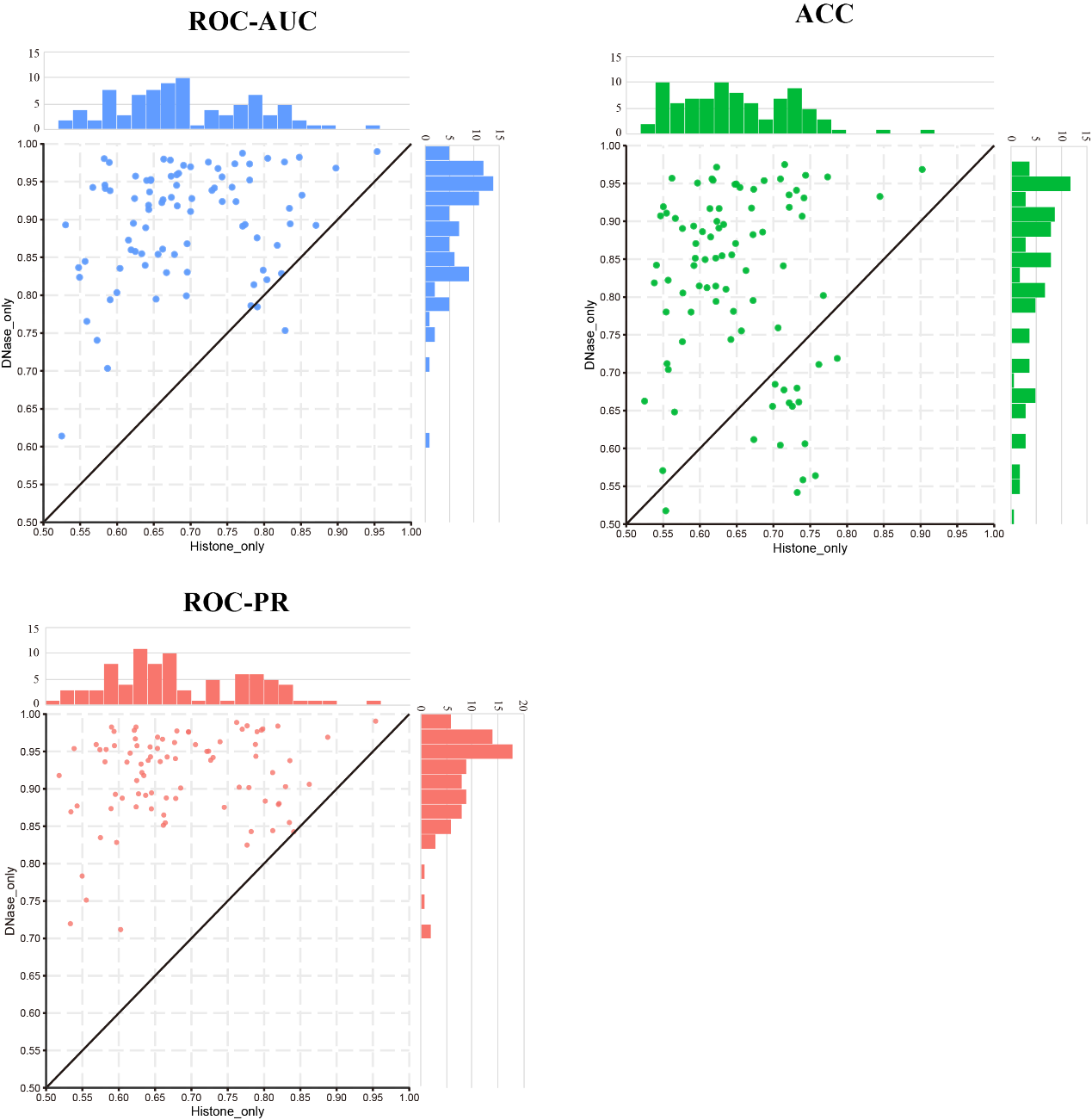

Supplement: Supplementary file 1 [file genes-13-01952-s001.zip › Figure S4.tif]

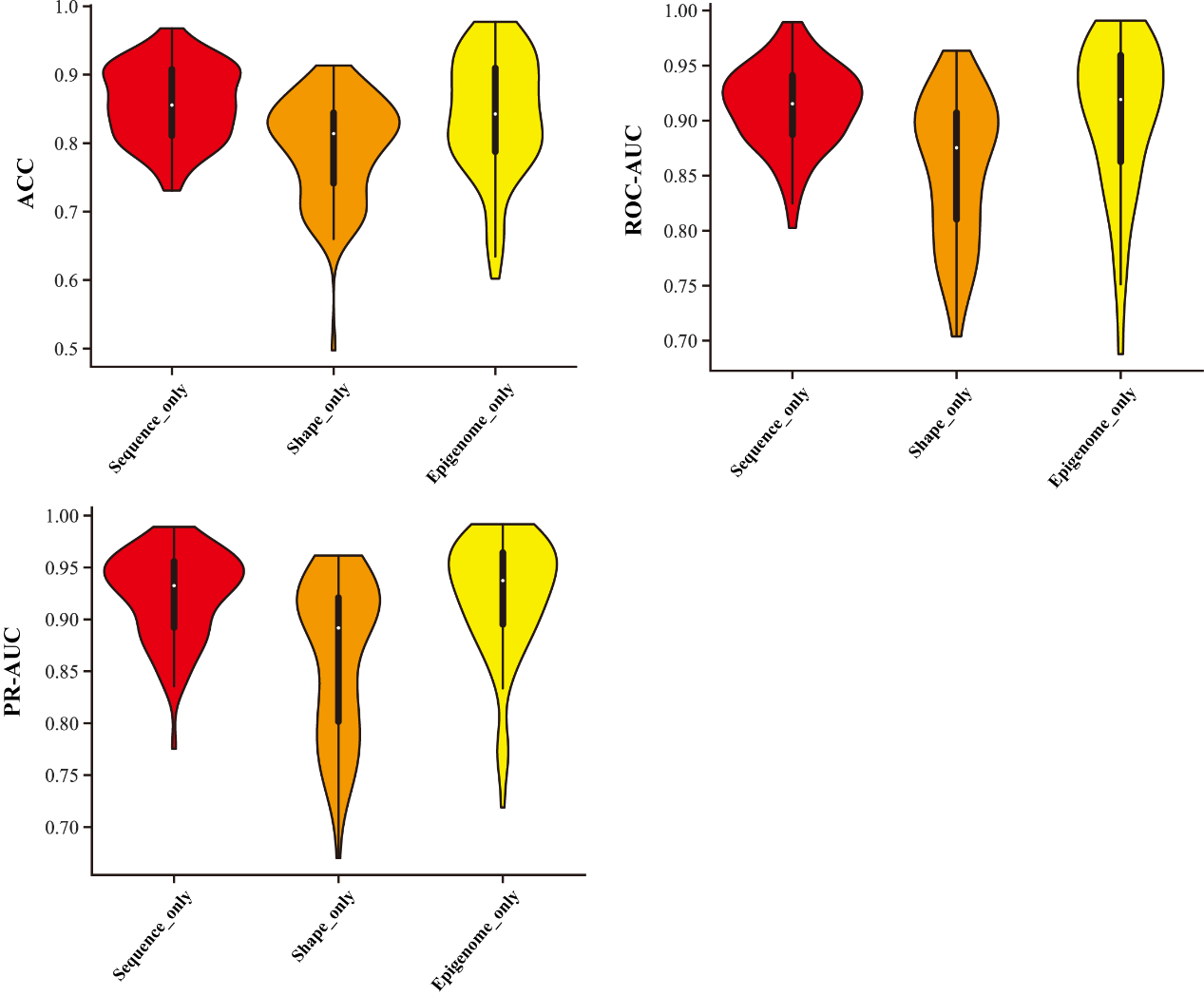

Supplement: Supplementary file 1 [file genes-13-01952-s001.zip › Figure S5.tif]

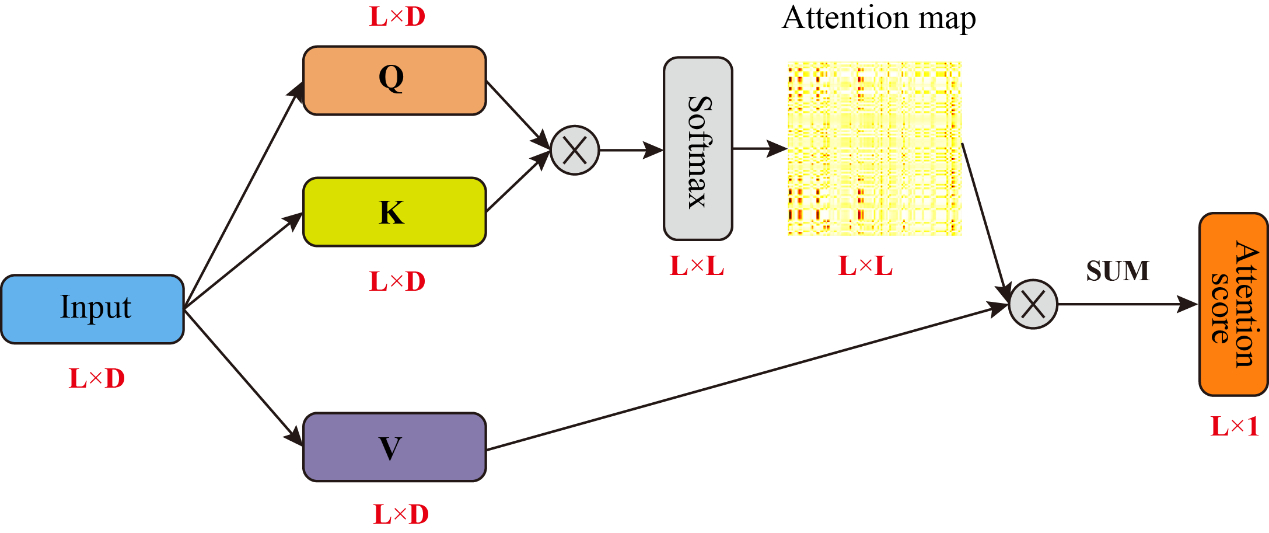

Supplement: Supplementary file 1 [file genes-13-01952-s001.zip › Figure S6.tif]

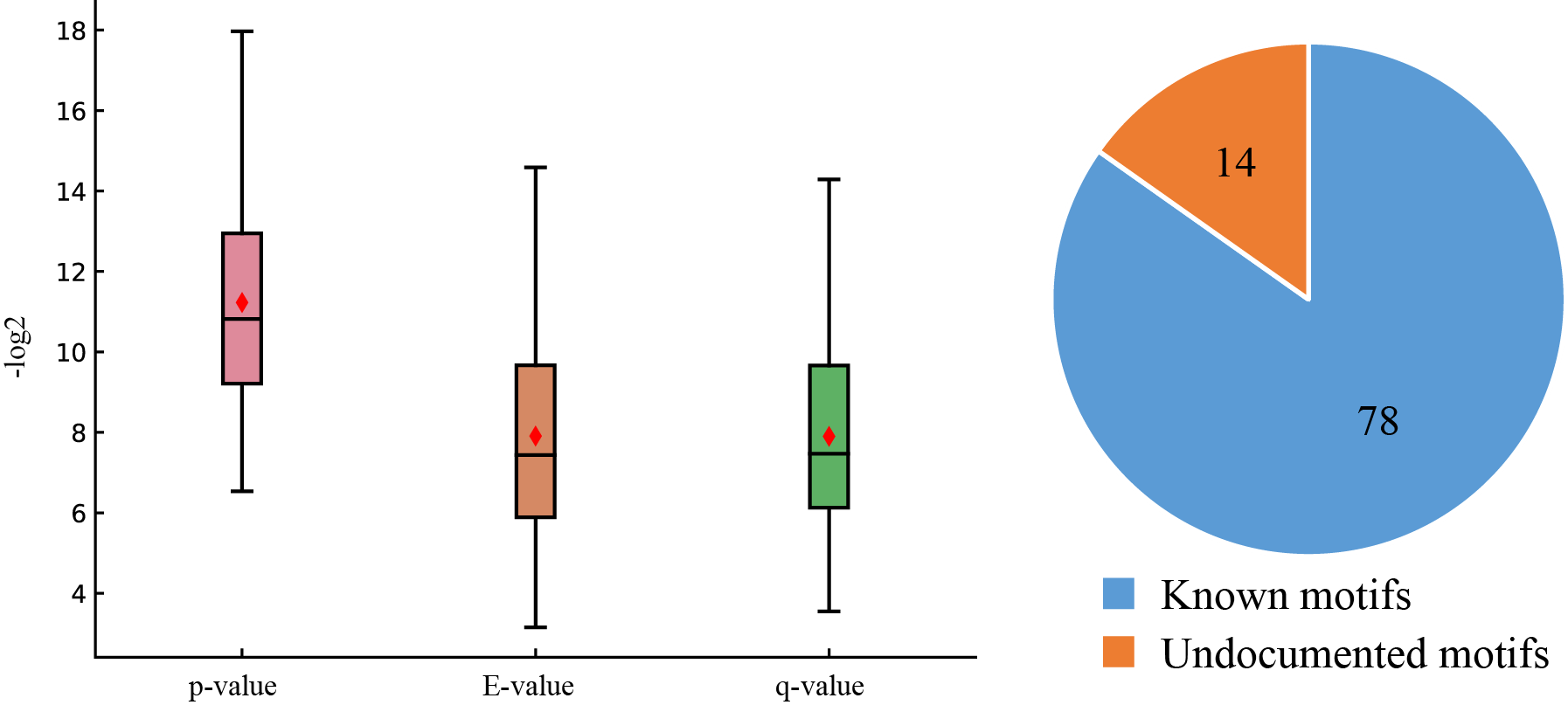

Supplement: Supplementary file 1 [file genes-13-01952-s001.zip › Figure S7.tif]

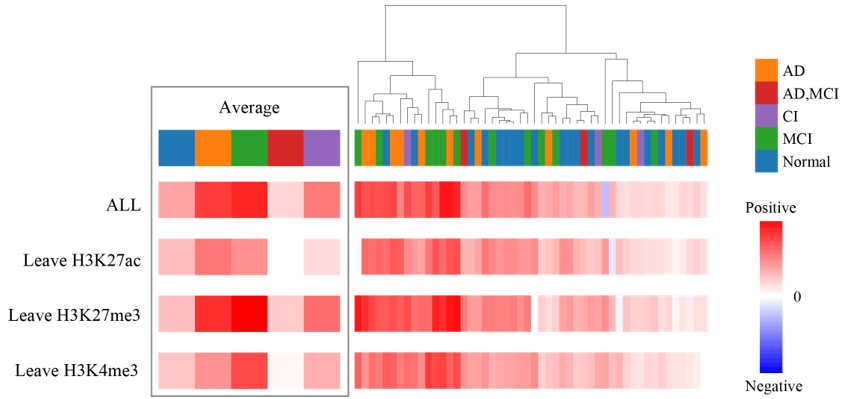

Supplement: Supplementary file 1 [file genes-13-01952-s001.zip › Figure S8.tif]

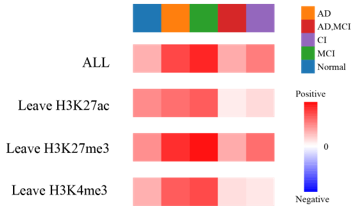

Supplement: Supplementary file 1 [file genes-13-01952-s001.zip › Figure S9.tif]
